# Comparing chatbots to psychometric tests in hiring: reduced social desirability bias, but lower predictive validity

**DOI:** 10.3389/fpsyg.2025.1564979

**Published:** 2025-04-25

**Authors:** Danilo Dukanovic, Dario Krpan

**Affiliations:** Department of Psychological and Behavioural Science, The London School of Economics and Political Science, London, United Kingdom

**Keywords:** AI, chatbots, hiring, professional selection, social desirability bias, big five, personality

## Abstract

This paper explores the efficacy of AI-driven chatbots in accurately inferring personality traits compared to traditional psychometric tests within a real-world professional hiring context. The study is driven by the increasing integration of AI tools in recruitment processes, which necessitates a deeper understanding of their reliability and validity. Using a quasi-experimental design with propensity score matching, we analysed data from 159 candidates and other professionals from Serbian and Montenegrin regions who completed both traditional psychometric assessments and AI-based personality evaluations based on the Big Five Personality model. A novel one-question-per-facet approach was employed in the chatbot assessments with a goal of enabling more granular analysis of the chatbot’s psychometric properties. The findings indicate that the chatbot demonstrated good structural, substantive, and convergent validity for certain traits, particularly Extraversion and Conscientiousness, but not for Neuroticism, Agreeableness, and Openness. While robust regression confirmed that AI-inferred scores are less susceptible to social desirability bias than traditional tests, they did not significantly predict real-world outcomes, indicating issues with external validity, particularly predictive validity. The results suggest that AI-driven chatbots show promise for identifying certain personality traits and demonstrate resistance to social desirability bias. This paper contributes to the emerging field of AI and psychometrics by offering insights into the potential and limitations of AI tools in professional selection, while developing an approach for refining psychometric properties of AI-driven assessments.

## 1 Introduction

The rapid implementation of Artificial Intelligence (AI) in recruitment has transformed how organisations identify, assess, and select candidates. Companies such as Pymetrics, Unilever, and Sapia have integrated AI-driven solutions to optimise hiring processes, reduce biases, and enhance the candidate experience (Dai et al., 2022; George et al., 2021). However, the fast adoption of these technologies has resulted in a significant gap between practical applications and academic validation—a *practitioner-academia gap* (Will et al., 2022).

Historically, psychometric tests have been the gold standard in assessing candidates’ personality traits, with a track record of predictive validity and reliability (Schmidt and Hunter, 1998). In contrast, AI’s role in hiring is relatively new, and its effectiveness, particularly in personality assessment, is an emerging area of research (George et al., 2021; Will et al., 2022). This study aims to bridge this gap by rigorously comparing AI-driven chatbots with traditional psychometric tests in inferring candidates’ personality traits.

Drawing inspiration from Fan et al. (2023), who introduced an approach to evaluate psychometric properties of AI methods, this study adopts a facet-level inference strategy to assess the AI chatbot’s performance. By integrating both traditional psychometric methods and AI chatbots in a practical, real-world selection scenario, this research seeks to contribute to existing literature and industry practises. Specifically, the study explores whether AI chatbots can accurately infer personality traits and mitigate social desirability bias in professional selection settings. By addressing this question, the study aims to offer a balanced perspective that integrates scientific rigour with practical relevance, ultimately contributing to the development of AI tools that are both validated by academic standards and applicable in real-world hiring processes.

The study introduces two significant expansions of Fan et al.’s (2023) work: enhancing ecological validity by applying the methods in a real-world hiring context and refining the methodological approach by using one open-ended question per personality facet. These innovations aim to improve the precision of personality assessments and open a path to systematic incremental improvement of chatbots. Importantly, this research on AI-based psychometrics is conducted in non-WEIRD (Western, Educated, Industrialised, Rich, and Democratic) countries (Serbia and Montenegro), where this emerging type of studies has been neglected. This context highlights the importance of exploring how long-standing methodologies such as traditional tests intersect with emerging AI-technologies, particularly in the setting where such approaches remain underexplored.

### 1.1 Psychometric testing in hiring

The hiring process evolved over time, incorporating various methods to effectively assess and select candidates. One approach that has consistently demonstrated strong predictive power, reliability, and validity over decades of research is psychometric testing. These tests are designed to measure various psychological attributes that predict job performance, including intelligence, personality traits, and behavioural tendencies.

Based on 85 years of research, Schmidt and Hunter (1998) found that psychometric tests, including cognitive tests and personality questionnaires, are highly effective in predicting job performance. These tests have a predictive validity of 0.67, indicating a strong correlation between test scores and job performance. This makes them the most powerful method in predicting job success, outperforming other approaches such as work sample tests and structured interviews.

A study in the retail sector by Chipana-Castillo et al. (2021) confirmed these findings, showing that psychometric tests are not only effective in predicting job performance but also in reducing turnover rates. Additionally, personality assessments, such as the Big Five Inventory (BFI), provide valuable insights into candidates’ behavioural tendencies and interpersonal skills. A meta-analytic review by Vinchur et al. (1998) highlighted that Extraversion and Conscientiousness, as measured by the BFI, are particularly strong predictors of sales performance, with corrected validity coefficients of 0.22 and 0.31, respectively.

The established reliability and validity of psychometric tests in predicting job performance have led to their widespread adoption in recruitment practises. This approach enables organisations to make informed and objective hiring decisions, contributing to their overall success.

### 1.2 Everybody lies: the issue of social desirability

Despite the success and validity of psychometric testing, a significant challenge remains: the possibility for respondents to manipulate their scores on self-reported measures. This phenomenon, known as *social desirability bias*, refers to the tendency of respondents to give socially desirable answers instead of choosing those that reflect their true feelings (Grimm, 2010).

In high-stakes professional selection processes, candidates are particularly motivated to present themselves in a favourable light. Job applications often nudge individuals to engage in response distortion to increase their chances of being selected (Ziegler and Kemper, 2013), leading to social desirability bias in various ways. For example, candidates may exaggerate positive traits or downplay negative ones to align with perceived desirable characteristics for the role, potentially compromising the validity of the assessment.

Selecting a candidate based on distorted responses can lead to suboptimal hiring decisions. Clarke and Robertson (2008) noted that response distortion may result in the selection of individuals who are not genuinely suited for the role, potentially increasing turnover rates and decreasing job performance, ultimately affecting both employers and the employees.

To mitigate the impact of social desirability bias, various methods have been developed, such as lie scales to detect response distortion (i.e., Crowne and Marlowe, 1960). However, the effectiveness of these methods remains subject to debate. While they are effective in identifying the presence of social desirability bias, they are limited to detection alone. Precisely inferring personality traits despite respondents’ social desirability bias is often unavailable, unreliable, or overly complex and demanding in practical settings, using methods such as the Multitrait-Multimethod matrix (Campbell and Fiske, 1959).

Mikulić and Sekulić (2014) explored the use and validity of lie scales in selection assessment settings. They highlighted that socially desirable responding is not merely a measurement error but may also reflect stable personality traits such as obedience or social conformism. This dual nature of socially desirable responses complicates the interpretation of high scores on lie scales, as it becomes challenging to distinguish between intentional response distortion and inherent personality characteristics. The study (Mikulić and Sekulić, 2014) also found that candidates with higher cognitive abilities scored lower on lie scales compared to those with lower cognitive abilities. Similar findings have been reported elsewhere. In this context, Furnham (2002) suggests that individuals with higher cognitive abilities are better at detecting and navigating these items, thereby presenting themselves more convincingly. In contrast, those with lower cognitive abilities may score higher due to defensiveness or naivety. Nevertheless, all candidates tend to engage in more socially desirable responding in selection contexts than in non-selection ones, raising questions about the appropriateness of lie scales for hiring decisions (Mikulić and Sekulić, 2014).

### 1.3 How well can AI infer personality traits?

Questions have arisen about whether AI capabilities can enhance the accuracy of personality assessments by identifying and adjusting for response biases more effectively than traditional methods. AI-driven tools, with their ability to analyse vast amounts of data and detect subtle patterns, may offer new ways to discern authentic personality traits, even in the presence of socially desirable responding.

However, while AI can enhance efficiency and objectivity, it can also perpetuate existing biases if not carefully designed and monitored (George et al., 2021; Will et al., 2022). To accurately evaluate the capabilities of novel AI methods—particularly their potential to mitigate social desirability bias—these methods must undergo rigorous validation processes comparable to those established for traditional psychometric tests over decades. This presents a significant challenge at the intersection of psychological and behavioural science and AI, as large language models (LLMs) are inherently stochastic and often operate as *black boxes*. This lack of transparency conflicts with the methodological rigour that underpins psychological and behavioural science (Mills, 2021).

Recent literature has begun to address these challenges, offering novel methods and frameworks. Will et al. (2022) introduced the HIRE framework, designed to evaluate the use of AI in hiring processes by focusing on aspects such as efficacy and user perception. This framework facilitates the comparability of AI tools and assess whether they make better, equal, or worse hiring decisions than humans. In addition, Grunenberg et al. (2024) conducted a study evaluating the psychometric properties of personality traits inferred from resumes and short text responses using machine learning algorithms. Their findings demonstrated that these AI models could predict the BFI personality traits with moderate accuracy, often surpassing traditional recruiter judgements.

Further research has shown promise in using AI to infer personality traits. For example, Jang et al. (2022) developed a study protocol using Natural Language Processing (NLP) to predict personality and psychological distress. By analysing text from semi-structured interviews based on the Five-Factor Model, they demonstrated the potential of NLP to provide insights into the understanding of personality through linguistic data. Moreover, Dai et al. (2022) provided another significant contribution by developing InterviewBERT, a specialised NLP model fine-tuned with a large corpus of interview responses. Their study demonstrated that textual content of interview answers can be used to reliably infer personality traits. The relevance of this study is not limited to theoretical findings, as InterviewBERT is already in practical use as part of AI-based recruitment solutions offered by Sapia.ai (2023),^1^ a US-based unicorn company specialising in AI-driven recruitment interviews.

Perhaps most relevant to the intersection of psychometrics and AI, Fan et al. (2023) proposed a method to evaluate the psychometric properties of personality traits inferred from AI-driven chatbot interactions. Using a sample of undergraduate students, the study assessed psychometric properties of personality scores derived from free-text responses during online conversations with a chatbot. They introduced a facet-based inference approach, which infers specific aspects of personality traits through interactive conversations.

A facet-based approach involves breaking down a personality trait into its facets (subcomponents). A chatbot then infers scores for these subcomponents, enabling the evaluation of its psychometric properties, which means that the chatbot is appraised as a psychometric instrument. The findings demonstrated that machine-inferred scores had acceptable reliability, good internal consistency, strong factorial validity, and moderate convergent validity, although discriminant validity was weaker. Notably, the scores showed incremental validity over self-reported measures, particularly in predicting outcomes such as GPA and peer-rated college adjustment. The study suggested that AI chatbots can complement traditional self-report measures in assessing personality traits.

Overall, studies by Fan et al. (2023), Grunenberg et al. (2024), and Dai et al. (2022), have shown promising results. However, AI-inferred and traditional measures remain only moderately aligned, with correlations typically ranging from 0.3 to 0.5. Certain traits, such as Agreeableness and Neuroticism, exhibit even weaker psychometric qualities and validity in AI inferred measures. Therefore, even if AI-driven approaches present a scalable and efficient alternative to traditional psychometric methods, ongoing research, rigorous validation, and methodological refinements are necessary to improve their robustness and applicability.

### 1.4 Evaluating psychometric properties of an AI chatbot

In the method proposed by Fan et al. (2023), they build upon Bleidorn and Hopwood's (2019) framework, which proposes three general classes of evidence for the validity of machine-inferred personality scores, based on Loevinger’s (1957) original model. These classes include: (1) substantive (content) validity; (2) structural validity; and (3) external validity.

Substantive validity refers to the degree to which test items adequately represent the construct being measured (Wijsen et al., 2022). Establishing substantive validity for machine-inferred personality scores is particularly challenging due to the data-driven nature of machine learning (ML) approaches, which rely on empirically identified features (Hickman et al., 2022). These features are often diverse and heterogeneous, making it difficult to determine *a priori* which features should predict specific personality traits. While some studies, such as Kosinski et al. (2013), have made progress in this area, the overall evidence remains limited.

Structural validity focuses on the internal characteristics of test scores, such as reliability, generalizability, and factorial validity (Wijsen et al., 2022). Reliability reflects the consistency of the scales in measuring the same constructs (Cohen and Swerdlik, 2018), whereas generalizability examines whether the model produces comparable personality scores across different contexts or samples. Finally, factorial validity evaluates whether machine-inferred scores can replicate established factor structures, such as the Big Five (Costa and McCrae, 2008; Goldberg, 1993). Literature demonstrates that AI-inferred scores consistently replicate the expected five-factor structure (Fan et al., 2023; Dai et al., 2022).

External validity is the extent to which the findings or measurements of a study generalise to other contexts, populations, or settings beyond the study conditions (Shadish et al., 2002). Its estimates refer to the relationships between scores and external, theoretically relevant variables, assessing convergent, discriminant, criterion-related, and incremental validity. Convergent validity refers to the degree of correlation between machine-inferred and questionnaire-derived scores, reflecting their agreement in measuring the same construct (Fan et al., 2023). In contrast, discriminant validity ensures that the measure effectively differentiates between distinct constructs. Moreover, criterion-related validity evaluates the relationship between personality scores and relevant external outcomes, such as academic performance. Incremental validity assesses whether machine-inferred scores enhance predictive power beyond that provided by traditional measures. Depending on the source of data, ranging from resumes and social media data to open-ended interview questions, personality scores inferred via machine learning have typically shown moderate criterion related validity (*r* = 0.2–0.5) (Fan et al., 2023; Dai et al., 2022; Grunenberg et al., 2024). Similarly, they have demonstrated moderate predictive validity. For example, Grunenberg et al. (2024) reported comparable or superior predictive accuracy to self-reported measures for job-related criteria such as vocational interests. Moreover, Fan et al. (2023) reported low criterion validity but noted incremental utility for outcomes such as GPA and peer adjustment. However, Orrù et al. (2020) raised concerns about generalizability and ethical issues, particularly for models relying on social media data.

### 1.5 Research question and hypothesis

Overall, building on the main insights from the reviewed literature, the present study advances the field by addressing key gaps in the validation and application of AI-driven personality assessments in recruitment. While prior research has demonstrated the potential of AI models (e.g., Fan et al., 2023; Dai et al., 2022), limitations remain in their practical relevance, particularly in high-stakes, real-world hiring scenarios. Additionally, challenges such as moderate alignment with traditional psychometric measures and weaker validity for specific traits like Agreeableness and Neuroticism highlight the need for further refinement. To address these gaps, the central research question of this study is whether AI chatbots can effectively infer personality traits without social desirability bias in professional selection contexts. We answer this question by adopting the method proposed by Fan et al. (2023) and applying it in the practical, real-world hiring scenario.

In this context, we propose the following hypotheses, drawing on insights from prior studies and theoretical frameworks:

*H1:* We anticipate replicating the findings of Fan et al. (2023), confirming that chatbot-inferred personality scores align with the Five-Factor structure and demonstrate robust psychometric properties. This includes substantive, structural, and external validity, as outlined in Bleidorn and Hopwood's (2019) framework.

*H2:* Candidates (experimental group), matched to non-hiring professionals (control group) using propensity scores, will demonstrate more socially desirable responses in traditional psychometric tests but not in chatbot-inferred personality assessments. Consistent with prior findings that showed candidates distort their responses on self-report measures (Mikulić and Sekulić, 2014; Ziegler and Kemper, 2013), we expect this to manifest as higher social desirability scores and exaggerated traits, such as heightened conscientiousness, in professional selection settings on self-reported measures from traditional tests. However, chatbot-inferred scores are hypothesised to be less susceptible to social desirability bias than traditional self-reports. This hypothesis is supported by findings from Fan et al. (2023), who demonstrated that chatbots provide incremental validity over traditional psychometric tests. Their research suggests that chatbots capture unique variance from conversational interactions, which may reflect more authentic expressions of personality. These interactions allow chatbots to detect subtle behavioural patterns that are less prone to distortion or faking, offering a potentially more reliable assessment of personality traits compared to self-report measures.

## 2 Methods

### 2.1 Research design

The study employs a quasi-experimental research design with group membership as the independent variable, distinguishing between two distinct groups: (1) professionals who are not in a hiring setting (control group) and (2) candidates participating in the hiring process (experimental group). Both groups completed psychometric questionnaires and interacted with an AI chatbot designed to infer personality traits. As prior literature showed, candidates exhibit significantly higher social desirability in comparison to respondents who are not in professional selection condition (Ziegler and Kemper, 2013; Mikulić and Sekulić, 2014). Therefore the control group in our research design provides a baseline for understanding how responses may be influenced by the selection environment, while the experimental group represents the practical application of psychometric testing and AI chatbot assessments in real-world recruitment scenarios.

### 2.2 Variables and instruments

#### 2.2.1 Main variables

##### 2.2.1.1 Big five

The Big Five model (Costa and McCrae, 2008), consisting of five major personality traits—*Extraversion*, *Agreeableness*, *Conscientiousness*, *Neuroticism*, and *Openness to Experience*—is chosen for its wide acceptance and use in both academic and practical settings, ensuring the alignment of the present study with existing literature. This model is well-validated and has been extensively used to predict job performance (Chipana-Castillo et al., 2021; Schmidt and Hunter, 1998; Vinchur et al., 1998). It is also prevalent in recent studies on AI applications in personality assessment that underpin this research, such as Fan et al. (2023), Grunenberg et al. (2024), and Dai et al. (2022).

We assessed participants’ BF scores using two instruments. As the traditional psychometric measure, we used the short 50-item Serbian version of the Big Five plus Two scale (Colovic et al., 2014) that measures the five BF traits using a Likert scale from 1 = “*strongly disagree*” to 5 = “*strongly agree*.” Moreover, we used a custom developed chatbot.

The chatbot was built on the no-code platform Fabrile (2025),^2^ created in collaboration between Ingram Technologies (2025),^3^ Montenegrin HR tech startup Recrewty (2025),^4^ and the authors of this paper. Fabrile was selected for its customization capabilities and the unique opportunity to develop a chatbot in the Montenegrin/Serbian language, which was unavailable on platforms like Juji.io, used in Fan et al.'s (2023) study. This customization ensured that the chatbot could infer personality traits based on the BFI model, tailored to the linguistic and cultural context of the study population.

To measure personality traits, the chatbot employed a novel question-per-facet approach, an extension of recommendations by Fan et al. (2023). Specifically, the chatbot asks an open-ended question targeting a specific facet and infers a score for that facet based on the respondent’s answer. This approach ensures a more granular analysis of psychometric properties by evaluating each facet individually, enabling the development of precise, and targeted interventions to improve question content. Unlike prior studies (Dai et al., 2022; Fan et al., 2023), which analysed the entire text for all traits simultaneously, this method focuses on facet-level responses, enhancing both the accuracy and the actionable insights of the personality assessment. The facets were defined following the framework of Smederevac et al. (2024), and each trait was measured as follows:

- *Extraversion*: sociability, assertiveness, and energetic level.
- *Agreeableness*: compassion, respectfulness, and trust.
- *Conscientiousness*: organisation, productiveness, and responsibility.
- *Neuroticism*: anxiety, depression, and emotional stability.
- *Openness to experience*: creative imagination, intellectual curiosity, and aesthetic sensitivity.

For each facet, the chatbot was programmed to ask one open-ended question, followed by a secondary prompt if the initial response was irrelevant or insufficiently detailed. As is standard in the AI and psychometric literature (Dai et al., 2022; George et al., 2021; Fan et al., 2023), the AI model infers personality traits and facets by analyzing and scoring the linguistic patterns in the open-text responses provided by the respondent. A transcript sample and the specific questions for each facet can be found in the Supplementary Material (see *Instruments used in the study* section, p. 4–14).

One limitation of the chatbot development process was the absence of an initial large-scale training. Since this was the first study of its kind in the Western Balkan region, it was important to assess whether the chatbot has the potential to measure the Big Five. So instead, the chatbot was fine-tuned using existing data and built on the extensive training of OpenAI’s ChatGPT 4.0 API (OpenAI, 2023). This approach relied on leveraging pre-trained AI models to maintain predictive accuracy and validity. Future studies should further refine the chatbot’s performance by training it on a large, domain-specific sample.

##### 2.2.1.2 Social desirability

Social desirability bias among participants was measured in two ways. First, we utilised the short 13-item version of the Marlowe-Crowne Social Desirability Scale (MC scale; Crowne and Marlowe, 1960; Reynolds, 1982). This scale assesses the tendency to respond in culturally approved but unlikely ways on binary Correct/False items. The purpose of including social desirability as measured by the MC scale was to establish that being in the professional selection group (treatment) induces socially desirable responding, which can distort self-reported scores on traditional psychometric traits.

The scale was translated into Montenegrian/Serbian language using the standard double translation method to ensure accuracy and cultural relevance (Sousa and Rojjanasrirat, 2011). Second, the Big Five personality traits model was used. This approach goes beyond the MC scale by assessing how participants respond to socially desirable traits such as Conscientiousness and Agreeableness (Ones and Viswesvaran, 1998).

For the traditional psychometric testing of the social desirability bias as part of Hypothesis 2, both measures were used. In contrast, for the social desirability testing using the chatbot, only the second approach (i.e., detecting the bias using the Big Five) was used, since the chatbot was specifically trained to predict the Big Five traits.

#### 2.2.2 Demographic variables

The study collected participant data on gender (male vs. female), age, education (primary education; high school; HND; bachelor’s degree; master’s Degree; PhD), job position (Entry level; Medior; Senior; Management; Executive and Other) and Industry.

Age, gender, education, and job position level served as benchmarks to validate the psychometric properties of the chatbot (Hypothesis 1: external validity). Specifically, age and gender were examined to ensure that the personality scores captured meaningful, real-world demographic differences, consistent with established findings in personality psychology. Education and job position were included to assess whether the scores correlated with real-world outcomes (predictive validity), such as professional roles and qualifications. More detailed information on demographics is presented in Table 1.

**Table 1:** Sample demographics.

Variable	Statistic	Entire sample *N* = 159	Control group *N* = 114	Candidates *N* = 45
Age	Mean	38.83	37.07	43
	Std. dev.	9.85	10.56	5.79
	Min	20	20	27
	Max	57	56	57
Gender	Male (1)	48	36	12
	Female (2)	109	76	33
	Other (3)	2	2	0
Education	High School (2)	17	12	5
	HND (3)	14	3	11
	Bachelor’s (4)	66	42	24
	MSc (5)	60	55	5
	PhD (6)	2	2	0
Industry	Banking/Finance	57	12	45
	IT/Telecom	28	28	0
	Government/Legal	12	12	0
	Health	4	4	0
	Education/Research	6	6	0
	Other	52	52	0
Job position	Entry level (1)	25	14	11
	Medior (2)	33	20	13
	Senior (3)	46	31	15
	Management (4)	27	27	0
	Executive (5)	11	11	0
	Other (6)	17	11	6

Values of coded variables in parenthesis.

When we wanted to test Hypothesis 2 and conduct propensity scores matchmaking followed by regression analysis, all five demographic variables were used as controls to account for systematic differences in personality assessments that could bias the comparison between control and experimental groups (Schmitt et al., 2008; Roberts et al., 2007).

### 2.3 Sample

#### 2.3.1 Determining sample size

Prior literature and G*Power statistical software were consulted to determine the optimal sample size. To evaluate psychometric properties of the chatbot, we aimed to use Confirmatory Factor Analysis (CFA). Kline (2015) suggested a minimum of 200 participants for simple models, while Hair et al. (2010) recommend a minimum sample size of 150 for models with strong loadings and simple structure. These guidelines provided a foundation and minimal requirement for the sample size.

To estimate the treatment effect of professional selection on social desirability, we opted for regression analysis and therefore used G*Power for sample size calculations (Faul et al., 2007). Based on prior research suggesting effect sizes on the border between moderate to large (Christiansen et al., 2010; Mikulić and Sekulić, 2014; Ones and Viswesvaran, 1998), we used an effect size (f^2^) of 0.30. Statistical power was set to 0.90 to reduce the risk of type II error (Borenstein et al., 2009; Cohen, 1998; Hedges and Rhoads, 2010; Kraemer and Thiemann, 1987), and the significance level (*α*) was set to 0.05. Finally, the number of predictors was set to six (one treatment and the five demographic variables used as controls). Based on these parameters, the required total sample size was 65 participants.

The final study sample had 159 participants, with 114 individuals in the control group and 45 in the candidate group (see Table 1 and section 2.3.2. below), which satisfied Hair et al. (2010) minimum requirements for CFA as well as the power calculations.

#### 2.3.2 Sampling and participants

The study was approved by the Research Ethics Committee of the authors’ University. Research took place in Serbia and Montenegro between 1st and 31st of July. These two countries share the same language and have strong cultural, historical, and business ties, which justifies their inclusion in a unified sampling approach.

Sampling for both the control and experimental group was conducted by the first author in collaboration with Recrewty, a Montenegrin HR Tech startup. Recrewty offered their clients and prospecting clients the opportunity to participate in this research. The incentives provided included free assessments based on psychometric tests and AI evaluations, as well as consultations for professional selections and employee training and growth, provided by Recrewty.

The goal was to gather a sample of professionals for control group, and candidates for experimental groups. To ensure the quality of the sample, professionals from various industries were targeted. In total, seven companies participated in the study, including five IT companies, one retail company, and one bank. The bank participated in both the control and hiring conditions, providing employee assessments for the control group and two professional selections (positions of Director of Exposition and Affluent Associate) for the experimental group. In contrast, the other six companies participated by including a portion of their employees in the assessment (control group).

The assessments were arranged in collaboration with the HR managers of these companies. Recrewty facilitated the data collection by sharing the results of the assessments and selections from their clients with the researcher. These collaborations were legally formalised by a consulting agreement between Recrewty and the companies, which allowed the data to be shared with the author for academic writing purposes. However, any other detail remained proprietary and owned by the companies.

To further enhance the sample size and ensure robust statistical power, additional participants were recruited via LinkedIn for the control group. This approach allowed the study to reach a broader audience and gather more diverse data. Overall, basic information for all recruited participants is presented in Table 1 while more detailed information and descriptive statistics can be found in Supplementary Table 1 (p. 15).

### 2.4 Procedure

For this research, we utilised Qualtrics (2025), an online survey platform (Qualtrics, Provo, UT), and a chatbot built on Fabrile, a no-code chatbot platform.

Participants entered the study via Qualtrics, where the first page presented the information and consent form, ensuring informed consent was obtained before proceeding with the survey. Participants then completed a survey containing questions about demographics, followed by short versions of the MC Scale (Crowne and Marlowe, 1960; Reynolds, 1982) and the BF Personality Questionnaire (Colovic et al., 2014). Upon completion of the survey, participants were redirected to the chatbot. To enable automatic redirection, we used JavaScript custom code in Qualtrics to generate a random 6-digit code in a *letter-number* format. Participants were instructed to copy and paste this code to ensure they remembered it. They were then redirected to the chatbot and asked to input their code.

The chatbot would not initiate interaction if the correct format of the code was not entered first. Once the code was confirmed, participants were asked a series of open-ended questions. Each question aimed to measure one facet of the Big Five personality traits, resulting in a minimum of 15 questions. If a participant’s response was irrelevant or too brief to infer a score, the chatbot would ask a follow-up question. At the end of the interaction, chatbot asked about the participant’s industry and role and gave brief qualitative feedback of results and recommendations for growth based on the assessment.

Whereas participants in the control group followed the procedure described above, those in the experimental group underwent a similar procedure, but within a more controlled environment. For these participants, two selection sessions, each lasting 1 h, were conducted on the 16th and 17th of July at 8 am (CET) for two bank roles: Director of Exposition and Affluent Associate. Secure Microsoft Teams meeting link from the bank was used to host the assessment. Candidates in the experimental group were informed about the nature of the testing via consent forms and by the company HR director and a representative from Recrewty. After completing the psychometric scales, participants were redirected to the chatbot, where they used their unique ID to initiate the interaction with the chatbot. This ensured that their responses can be linked to the test scores, while ensuring anonymity. They were told that their personality traits, measured by psychometric tests, would be used to create anonymized reports by the consultant. These reports, coded by unique ID, would include personality assessments, and suggest interview questions for later stages. Participants were assured that the assessment was informative and non-discriminatory, and they had the right to refuse participation at any moment without consequences.

## 3 Results

Data preparation was conducted using Microsoft Excel (Microsoft Corporation, 2023, Redmond, WA), and statistical analyses were performed using R Studio (RStudio Team, 2020). Microsoft Excel was chosen for convenience for screening, organising, and cleaning data. R Studio was selected for statistical analyses because it offers a comprehensive set of tools, including specific packages relevant to this study.

### 3.1 Data preparation

Firstly, the data from the chatbot scores needed to be matched with the data from the survey based on unique matching codes provided to participants. The survey received 264 responses; however, only 159 participants completed the interaction with the chatbot. Participant who did not finish the interaction were excluded from the sample.

The data was then scanned, cleaned, and checked for outliers and missing data in Microsoft Excel. During this process, it was identified that seven participants had entered their birth year instead of their age in the open response field for age. These entries were corrected by calculating the participants’ ages from their birth years. Additionally, there were 21 instances of missing responses on various items across different participants. To address this, the average value of the respective item was used to replace the missing values (Enders, 2010).

### 3.2 Preliminary analysis: testing psychometric properties of traditional tests (big five and Marlowe-Crowne scales)

To test Hypothesis 1 by evaluating psychometric properties of the chatbot, it was first necessary to confirm the structure of the traditional scales we used. For this purpose, we conducted a CFA using the *lavaan* package in R Studio (Rosseel, 2012). CFA is used to test whether data fits a hypothesised model, and whether the items appropriately measure the intended constructs (Brown, 2015; Kline, 2015).

For the BFI we used the Maximum Likelihood (ML) method. This estimator is suitable for continuous data and assumes multivariate normality, making it appropriate for the typically distributed data (i.e., normal distribution) in personality assessments (Brown, 2015). For the MC Scale, which has binary responses, we used the Diagonally Weighted Least Squares (DWLS) method. DWLS is ideal for ordinal or binary data as it does not assume continuous variables or normal distribution (Li, 2016; Muthén, 1993).

The CFA for the BFI confirmed the expected five-factor structure and demonstrated acceptable model fit and good reliability (Table 2). While Chi-square (*χ*^2^) value was significant, and Comparative Fit Index (CFI) and Tucker-Lewis Index (TLI) were slightly below conventional threshold, given the model complexity and sample size, these values are still acceptable (Kline, 2015). Furthermore, fit indices like Root Mean Square Error of Approximation (RMSEA) and Goodness of Fit index (GFI) are more reliable in this specific case, as they are less sensitive to sample size and provide a good measure of fit per degree of freedom (Browne and Cudeck, 1993). RMSEA was 0.074, indicating a reasonable error of approximation in the population and GFI was 0.968, indicating a good fit. Reliability coefficients were assessed using Cronbach’s alpha and indicated good internal consistency for all BFI scales (Table 2).

**Table 2:** Estimates for BFI and MC scales.

Estimate	BFI	Marlowe-Crowne
Chi-square (*χ*^2^)	2182.359***	86.258*
df	1,165	65
CFI	0.705	0.947
TLI	0.690	0.937
RMSEA	0.074	0.045
GFI	0.968	0.998
Cronbach’s alpha	0.737–0.881	0.728

df, degrees of freedom; *p < 0.05, **p < 0.01, ***p < 0.001; Cronbach’s alpha represents a range reflecting the values for the 5 factors.

Regarding the MC scale, we also confirmed the expected single-factor structure and demonstrated good model fit and reliability (Table 2). As for the BFI measure, the Chi-square (*χ*^2^) value was significant, whereas other, more relevant indices showed good (CFI = 0.947, TLI = 0.937; > 0.90) to excellent fit (GFI = 0.998). Cronbach’s alpha of 0.728 demonstrated decent internal consistency.

Overall, both scales replicated a typical pattern of findings from the literature.

### 3.3 Hypothesis 1: testing psychometric properties of the chatbot

Hypothesis 1, which tested the psychometric properties of AI-inferred personality traits, received mixed support across various validity metrics. Given the complexity and the scope of the findings, they are summarised in Table 3 and in the next paragraph, while the detailed analyses are reported and discussed in the following sections.

**Table 3:** Summary of findings for H1: psychometric properties of AI-inferred scores.

Validity type		O	C	E	A	N
Structural validity		Partially supported	Supported	Supported	Partially supported	Supported
Substantive validity		Supported	Partially supported	Supported	Partially supported	Supported
External validity	Convergent validity	Supported	Supported	Supported	Partially supported	Not supported
	Discriminant validity	Partially supported	Supported	Partially supported	Not supported	Not supported
	Criterion-related validity	Partially supported	Partially supported	Partially supported	Not supported	Partially supported
	Predictive validity	Not supported	Not supported	Not supported	Not supported	Not supported
	Incremental validity	Not supported	Not supported	Not supported	Not supported	Not supported

OCEAN refers to the Big Five personality traits model: O, Openness to Experience; C, Conscientiousness; E, Extraversion; A, Agreeableness; N, Neuroticism. Each trait represents a spectrum along which individual personality differences are measured.

Structural validity was generally supported, with acceptable fit indices for the hypothesised five-factor model and strong factor loadings for Extraversion, Neuroticism, and Conscientiousness, though Agreeableness and Openness showed weaker internal consistency and discriminant validity. Substantive validity was confirmed for most traits, particularly Extraversion and Neuroticism, but certain facets of Agreeableness and Conscientiousness demonstrated weaker item alignment. For external validity, convergent validity was supported for Extraversion, Conscientiousness, and Openness but was weaker for Agreeableness and absent for Neuroticism. However, discriminant validity issues emerged, particularly for Agreeableness and Neuroticism. Criterion-related validity was partially supported, with some traits (e.g., Conscientiousness) demonstrating significant differences across demographic groups, but predictive and incremental validity for career achievements were not supported. Traditional psychometric traits, particularly Conscientiousness and Openness, consistently outperformed AI-inferred scores in predicting education and job position levels.

#### 3.3.1 Structural validity

To assess the structural validity of the chatbot’s psychometric properties and to verify whether the observed facets align with the hypothesised five-factor structure, CFA was conducted.

Visual inspection of the data distribution (see Supplementary Figures 1–5, p. 20–22) indicated deviations from normality, prompting further examination. Univariate normality was assessed using the Shapiro–Wilk test, which revealed significant deviations across all traits (W = 0.77952–0.95345, *p* < 0.001). Multivariate normality tested using Mardia’s test with the *MVN* package (Korkmaz et al., 2014), also showed significant deviations (*p* < 0.001). Therefore, a robust CFA using the MLR estimator was employed, which is a good alternative when the assumptions for the *maximum likelihood* method are not met.

The model fit was assessed using several indices (Table 4). While Chi-square was significant [*χ*^2^(80) = 146.22, *p* < 0.001], we again focused on other more reliable estimates less impacted by sample size. The Robust CFI (0.905) and TLI (0.875) suggested an acceptable fit, while the RMSEA (0.078) indicated a moderate fit. Finally, GFI (0.880) demonstrated that the model explained a substantial portion of the data’s covariance. Table 5 further presents the standardised factor loadings, scale reliability (Cronbach’s alpha), and individual item reliability for each of the five latent constructs measured.

**Table 4:** Model fit indices (MLR estimator).

Fit index	Value	Interpretation
GFI	0.880	Acceptable fit
CFI	0.905	Acceptable fit
TLI	0.875	Acceptable fit
RMSEA	0.078	Moderate fit

**Table 5:** Factor loadings and reliability analysis.

Latent variable	Facet	Factor loading	Scale reliability (α)	Individual item reliability (r)
Extraversion	Sociability	0.813		0.70
	Assertiveness	0.750	0.80	0.63
	Energy level	0.720		0.62
Agreeableness	Compassion	0.554		0.32
	Respect	0.585	0.58	0.44
	Trust	0.561		0.46
Conscientiousness	Organisation	0.697		0.54
	Productivity	0.833	0.67	0.63
	Responsibility	0.484		0.38
Neuroticism	Anxiety	0.911		0.79
	Depressivity	0.814	0.85	0.73
	Emotional stability	0.716		0.66
Openness	Creative imagination	0.765		0.56
	Intellectual curiosity	0.640	0.64	0.47
	Aesthetic sensitivity	0.472		0.34

Regarding the latent constructs, extraversion showed strong factor loadings (0.720–0.813) and a Cronbach’s alpha of 0.80, indicating good internal consistency. Agreeableness had moderate loadings (0.554–0.585) and a lower alpha of 0.58, suggesting weaker internal consistency. Conscientiousness had mixed loadings, with Productivity at 0.833 and Responsibility at 0.484, but its alpha was 0.67, indicating acceptable reliability. Neuroticism had high loadings (0.716–0.911) and a strong alpha of 0.85 and Openness showed variable loadings, with higher loadings for Creativity and Curiosity (0.640–0.765) and lower for Aesthetic Sensitivity (0.472) alongside the alpha of 0.64, indicating moderate reliability.

Overall, the analyses confirmed the structural validity of the hypothesised five-factor model. The fit indices suggested that the model as a whole offers an acceptable representation of the underlying constructs, with certain areas, particularly Agreeableness and Openness, showing room for improvement. These results underscore the overall validity of the chatbot’s measurement structure, while also highlighting specific facets where refinement is necessary.

#### 3.3.2 Substantive (content) validity

Using *one question-per-facet* approach has enabled a valid check of substantive validity. To ensure that each facet accurately reflects its corresponding trait we checked facet-trait correlations and the discrimination index for each of the five traits (Haynes et al., 1995).

Facet-trait correlations were computed by correlating individual item scores with the total score for the corresponding trait. The results, presented in Table 6, demonstrate strong correlations for most traits, particularly for Extraversion, where correlations ranged from 0.81 to 0.89, indicating that the individual items are highly aligned with the overall trait score. Similarly, Neuroticism showed robust correlations, with values between 0.83 and 0.92, confirming that the items within these facets are measuring their intended constructs. However, for Agreeableness, the correlations were slightly lower, ranging from 0.60 to 0.82, suggesting that while the items are still reflective of the trait, there may be room for improvement.

**Table 6:** Facet-trait correlations.

Trait	Facet	Correlation
Extraversion	Sociability	0.89
	Assertiveness	0.83
	Energy Level	0.81
Agreeableness	Compassion	0.60
	Respect	0.78
	Trust	0.82
Conscientiousness	Organisation	0.87
	Productivity	0.81
	Responsibility	0.67
Neuroticism	Anxiety	0.92
	Depressivity	0.88
	Emotional stability	0.83
Openness	Creative imagination	0.83
	Intellectual curiosity	0.81
	Aesthetic sensitivity	0.64

Higher correlations indicate that individual items within a facet align well with the overall trait score, suggesting that the items effectively measure their intended constructs. Values closer to 1 demonstrate stronger relationships, while lower values may indicate items that are less consistent with the overall trait.

The discrimination index, which measures the difference in mean item scores between high and low scorers, tells us how well each item distinguishes between individuals with varying trait levels. The maximum possible score for the discrimination index depends on the scale and dataset but typically ranges from 0 to around 30 in many psychometric applications. Values near the upper end suggest excellent discriminative power, while lower values may indicate the need for item revision or removal (Crocker and Algina, 2006). As shown in Table 7, Extraversion, Neuroticism, and Openness exhibited strong discriminative power.

**Table 7:** Discrimination index results.

Trait	Facet	Discrimination index
Extraversion	Sociability	26.11
	Assertiveness	19.71
	Energy level	16.75
Agreeableness	Compassion	10.43
	Respect	14.87
	Trust	20.37
Conscientiousness	Organisation	19.23
	Productivity	12.51
	Responsibility	10.02
Neuroticism	Anxiety	21.82
	Depressivity	24.93
	Emotional instability	17.33
Openness	Creative imagination	19.57
	Intellectual curiosity	19.19
	Aesthetic sensitivity	13.66

The discrimination index reflects the extent to which an item differentiates between individuals with high and low scores on the overall trait. Higher values suggest that the item is more effective at distinguishing between individuals with differing levels of the trait, whereas lower values may indicate that the item has less discriminative power and may require further refinement.

Extraversion items had notably high discrimination indices, with Sociability at 26.11, Assertiveness at 19.71, and Energy Level at 16.75, indicating these items effectively differentiate between individuals with high and low Extraversion. Neuroticism also showed strong discrimination, particularly with Depressivity (24.93) and Anxiety (21.82), highlighting the effectiveness of these items in distinguishing varying levels of neuroticism.

Openness demonstrated good discrimination, especially in Creative Imagination (19.57) and Intellectual Curiosity (19.19). Aesthetic Sensitivity, however, had a lower index (13.66), suggesting moderate differentiation. Agreeableness and Conscientiousness displayed moderate discrimination indices, with items like Trust (20.37) and Organisation (19.23) performing well, while Compassion (10.43) and Responsibility (10.02) were less effective.

#### 3.3.3 External validity

Finally, to evaluate external validity, we accessed convergent, discriminant, criterion related and incremental validity, in line with recommendations by Fan et al. (2023) and Bleidorn and Hopwood's (2019).

##### 3.3.3.1 Convergent and discriminant validity

Following the approach used in Fan et al. (2023), to evaluate convergent and discriminant validity, we examined the correlations of AI-inferred scores with scores measured by a psychometric test. Ideally, each AI-inferred trait score should correlate strongly and significantly with its corresponding psychometric measure (indicating good convergent validity) and not with other traits (indicating good discriminant validity).

The analysis of convergent validity, which was assessed by examining the correlations between AI-inferred traits and their corresponding traditional psychometric measures (Table 8), yielded mixed results. Specifically, Extraversion and Conscientiousness demonstrated good convergent validity, with significant positive correlation (*r* = 0.443***; *r* = 0.449***). Openness also showed acceptable convergent validity with a correlation of 0.362 (*p* < 0.001). However, Agreeableness showed somewhat weaker convergent validity (*r* = 0.256***) and Neuroticism did not display a significant correlation (*r* = −0.099, *p* > 0.05), indicating a lack of convergent validity for this trait.

**Table 8:** Correlation matrix between psychometric and AI-inferred scores.

		Psychometric scores				
		O	C	E	A	N
AI inferred scores	O	0.362***	0.012	0.164*	0.009	−0.260***
	C	0.025	0.449***	0.300***	0.094	−0.221**
	E	0.238**	0.219**	0.443***	0.152	−0.321***
	A	0.237**	0.195*	0.342***	0.256***	−0.272***
	N	0.061	−0.128	−0.099	0.074	0.089

OCEAN refers to the Big Five personality traits model: O, Openness to Experience; C, Conscientiousness; E, Extraversion; A, Agreeableness; N, Neuroticism. Each trait represents a spectrum along which individual personality differences are measured. Cor. Two-tailed. Significance levels: *p < 0.05, **p < 0.01, ***p < 0.001.

However, results in Table 8 indicate issues with the discriminant validity of the AI-inferred trait scores. Most AI-inferred scales significantly correlate with Neuroticism, except for the AI Neuroticism scale. Moreover, the AI Agreeableness scale shows stronger correlations with Extraversion (*r* = 0.342***) and a negative correlation with Neuroticism (*r* = −0.272***) than with the psychometric Agreeableness measure (*r* = 0.256***). This implies that the AI Agreeableness scale is not specific enough and overlaps with other traits.

In conclusion, while some AI-inferred traits exhibit good convergent validity, the discriminant validity is problematic, particularly for Neuroticism and Agreeableness, where the measures appear to lack distinctiveness.

##### 3.3.3.2 Criterion-related validity (predictive and incremental validity)

To further evaluate external validity, we access criterion-related validity. The criterion-related validity of the AI-inferred traits was assessed through a series of analyses including *t*-tests, multinomial logistic regression, and logistic regression.

First, independent samples *t*-tests were conducted to explore whether there were significant differences in the AI-inferred traits (Extraversion, Agreeableness, Conscientiousness, Neuroticism, and Openness) between males and females. As in shown in prior cross-cultural large-scale studies and meta-analysis (Schmitt et al., 2008; Weisberg et al., 2011) it is expected that women score higher than men on Neuroticism, Agreeableness, Extraversion and Conscientiousness.

Two participants who identified as “Other” were excluded from this analysis because their category contained only two individuals, which is insufficient for meaningful statistical analysis. The results indicated that there were no significant differences in Extraversion, Agreeableness, or Openness between the two groups, with *p*-values of 0.2202, 0.4857, and 0.5282, respectively. However, significant differences were found for Conscientiousness and Neuroticism. Specifically, males scored significantly lower on Conscientiousness (= −2.8593, *p* < 0.01) and Neuroticism in comparison to females (= −2.8128, *p* < 0.01), confirming the pattern shown on traditional measures in prior studies.

Next, we aimed to test the predictive validity of AI-inferred personality scores by examining their ability to predict real-world career achievements, specifically focusing on two key outcomes: level of education and job position level. The primary goal was to determine whether AI-inferred scores could predict these meaningful and measurable indicators of career success akin to how traditional psychometric assessments were able to (Chipana-Castillo et al., 2021; Schmidt and Hunter, 1998; Vinchur et al., 1998). Furthermore, we investigated the incremental validity of AI-inferred traits when combined with traditional psychometric measures to understand whether they contribute additional explanatory power in predicting these outcomes. This approach follows the standard validation framework used in prior research (e.g., Fan et al., 2023), where new measures are first examined independently for predictive validity before assessing their incremental validity over established psychometric tools (Hunsley and Meyer, 2003).

Education variable was reclassified into three categories to ensure sufficient sample sizes within each group: Primary Education (combining Primary School and High School), Secondary Education (combining HND and bachelor’s degree), and Higher Education (combining master’s degree and PhD). Five multinomial logistic regressions with age, gender and professional selection conditions as controls, did not yield significant results. Across all traits, coefficients ranged from 0.003 to 0.051 with standard errors between 0.01 and 0.031, indicating weak and non-significant associations (see Supplementary Table 3, p. 16).

For a second analysis, binary dummy variable was created on job position: High-Level Roles (combining Management and Executive roles) and Other Roles (combining rest of the categories). Five logistic regressions with age, gender, and professional selection condition as controls, showed again no significant relationship across all five AI inferred scores (Supplementary Table 4, p. 16).

To assess the incremental validity of AI-inferred traits over traditional psychometric scores, we conducted multinomial logistic regressions for education level and logistic regressions for job role level, including both AI-inferred and traditional traits, with age, gender, and the selection condition as control variables (see Supplementary Tables 6, 7, p. 17–18).

For education level, adding AI-inferred traits did not significantly improve the prediction. Traditional psychometric measures, Conscientiousness emerged as significant predictor with higher education (compared to primary education).

For job role level, adding AI-inferred traits to the model did not significantly enhance predictive power [*χ*^2^ (5) = 1.03, *p* = 0.960]. None of the AI-inferred or Psychometric scores were significant predictors, although traditional Openness approached significance with a *p*-value of 0.08, indicating a potential, though not definitive, relationship.

Overall, the evaluation of the external validity of AI-inferred traits revealed a mixed picture. Criterion-related validity was partially supported: Extraversion and Conscientiousness demonstrated good convergent validity, while Neuroticism and Agreeableness revealed significant issues in that segment. For predictive validity, none of the AI-inferred traits significantly predicted career achievements (education level or job position), and they did not enhance the predictive power of traditional psychometric measures, indicating no incremental validity. Traditional psychometric traits, particularly Conscientiousness and Openness, remained stronger predictors of education level, though predictive validity for job position was inconclusive.

### 3.4 Testing hypothesis 2: social desirability bias

#### 3.4.1 Propensity score matching

To test Hypothesis 2, we aimed to compare the treatment and control groups on specific social desirability indicators assessed via traditional tests or the chatbot. To ensure comparability, participants were first matched based on propensity scores using the *MatchIt* package in R Studio (Grafer, 2023). Variables included in the matching process were gender, age, education, and job position. Industry was not included due to a large amount of missing data and the specific case of the treatment group being only from the banking sector while the control group was heterogeneous in industry composition.

First, we assessed the initial balance of the covariates between groups. The summary of balance before matching indicated significant imbalances across covariates (Table 9). The standardised mean difference (SMD) was 1.172 and empirical cumulative distribution function (eCDF) mean for the distance was 0.3176, highlighting significant imbalance (Table 9).

**Table 9:** Balance between the treatment and control groups across all participants.

Covariate	Means treated	Means control	Std. mean diff	eCDF mean
Distance	0.485	0.203	1.172	0.317
Gender	1.733	1.701	0.070	0.022
Age	43.288	37.07	1.074	0.177
Education	3.644	4.280	−0.766	0.127
Job position	2.622	3.298	−0.438	0.125

Gender was coded as 1 = Male, 2 = Female, 3 = Other. Education was coded as 1 = Primary School, 2 = High School, 3 = Higher National Diploma, 4 = Bachelor’s Degree, 5 = Master’s Degree (MSc), 6 = Doctorate (PhD). Job position was coded as 1 = Entry Level/Junior, 2 = Medior (Mid-Level), 3 = Senior, 4 = Management, 5 = C-Level (Executive), 6 = Other. A well-balanced match is indicated by covariate differences close to 0, minimising the likelihood of confounding the treatment effect.

We then applied *1:1 nearest neighbour matching without replacement* using *logistic regression (glm)* to estimate the propensity scores and explored different methods to determine the best balance-sample size trade-off, including using different calliper widths (0.2–0.25 as advised by Austin, 2011). Additionally, we explored the *full matching* method and *optimal* matching method, both of which are implemented in statistical software as predefined functions (Table 10). For the full matching method, we specified a *probit link function,* as it often improves covariate balance compared to logistic regression since it models probabilities using the cumulative normal distribution to better match treated and control groups under certain data conditions (Grafer, 2023).

**Table 10:** Results of the matching process.

Method	Matched treated	Matched control	Std. mean diff	eCDF mean
Nearest neighbour	45	45	0.386	0.311
0.2 calliper	28	28	0.087	0.142
0.25 calliper	33	33	0.047	0.090
Full method	20.06 (ESS)	45	0.015	0.088
Optimal method	45	45	0.386	0.311

Initial size of Treated = 45; Control = 114. A well-balanced match is indicated by covariate differences close to 0, minimising the likelihood of confounding the treatment effect.

**Figure 1:**
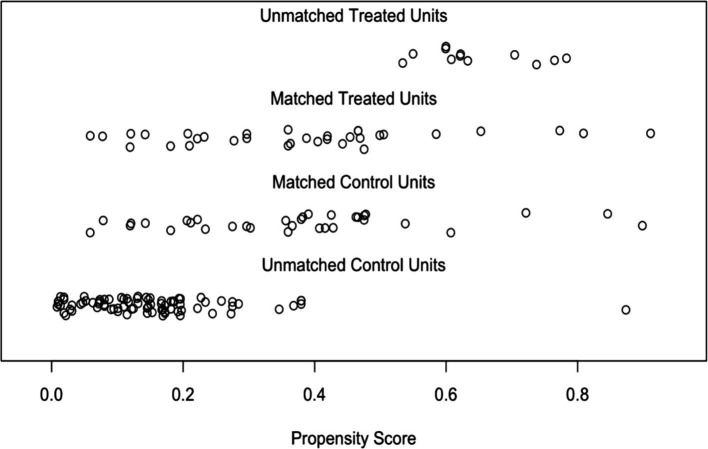
Distribution of the propensity scores.

Based on the balance improvement and the number of matched participants, we selected a calliper of 0.25. This choice ensured optimal sample size (33 matched pairs; 66 in total—aligned with the G*Power recommendation) while achieving excellent balance. For instance, the standardised mean differences for gender (−0.067), age (0.015), education (0.036), and job position (−0.059), as well as the total SMD (0.047) indicated an excellent balance (Table 11). The distribution of the propensity scores is illustrated in Figure 1.

**Table 11:** Summary of balance for matched data (Calliper = 0.25).

Covariate	Means treated	Means control	Std. mean diff	eCDF mean
Distance	0.383	0.373	0.047	0.090
Gender	1.722	1.757	−0.067	0.030
Age	43.424	43.333	0.015	0.242
Education	3.878	3.848	0.036	0.212
Job role lvl.	2.939	3.030	−0.059	0.212

#### 3.4.2 Regression analysis on psychometric test measures

A linear regression analysis was conducted on a sample of 66 matched participants based on propensity scores to estimate the average treatment effect (ATE) of being in a professional selection group on social desirability, as assessed using the MC scale and the BF traits approach. This analysis aims to test whether professional selection conditions lead to an increase in social desirability bias (MC scale), which in turn may systematically inflate or distort self-reported personality traits measured via traditional psychometric tests.

Regression was chosen over ANOVA to enable the estimation of ATE and the inclusion of control variables, providing a clearer estimate of the treatment effect in this quasi-experimental study.

Six separate regression analyses were conducted in parallel, with the MC scale score and each of the five BFI traits serving as dependent variables in individual models (for descriptive statistics, see Supplementary Table 1, p. 15). We additionally included covariates from propensity scores matching (i.e., age, gender, education, and job position) to control for any possible additional confounds. To account for potential industry-specific differences, a binary dummy variable was created for the banking industry versus other industries and included as a control.

Regression results (Table 12) showed that the treatment variable is significantly associated with MC score and four BFI scores, indicating that being in the professional selection group impacts these scores. However, to assess the robustness and validity of these results, several checks had to be performed, including linearity, normality of residuals, multicollinearity, and autocorrelation. Firstly, Durbin-Watson test indicated significant autocorrelation for MC scale (DW = 0.516, *p* < 0.000) and Agreeableness (DW = 1.513, *p* < 0.05), which necessitated the use of HAC (heteroskedasticity and autocorrelation consistent) standard errors to mitigate this issue (Newey and West, 1987).

**Table 12:** Regression estimates for professional selection on psychometric test measures.

	(1) MC	(2) O	(3) C	(4) E	(5) A	(6) N
Panel A: linear regression						
Professional Selection	4.589*** (1.027)	0.448* (0.597)	0.460* (0.214)	0.605** (0.212)	0.650* (0.274)	−0.218 (0.247)
Constant	3.183 (3.091)	2.812*** (0.597)	3.558*** (0.646)	3.586*** (0.638)	3.176*** (0.274)	2.201** (0.745)
Panel B: HAC standard errors						
Professional Selection	4.553*** (1.203)				0.640*** (0.173)	
Constant	3.550* (1.745)				3.066*** (0.679)	
Panel C: regression with squared dependent variables						
Professional selection		3,158* (1.552)		4.774** (1.630)		
Constant		8.110 (3.158)		13.131** (4.906)		
Panel D: robust regression						
Professional Selection			0.526* (0.253)			
Constant			3.984*** (0.502)			

Marlowe-Crowne social desirability bias (MC) and the Big Five Inventory (BFI) traits: openness (O), conscientiousness (C), extraversion (E), agreeableness (A), and neuroticism (N). Standard errors are reported in parentheses. Significance Levels: *p < 0.05; **p < 0.01; ***p < 0.001. All models include controls for gender, age, education, job position, and industry type. The sample size for all models is 66. In alignment with prior studies, average scores for the BF personality traits were calculated based on responses on corresponding items. For the Social Desirability (MC) scale, scores were derived by summing responses across 13 items, with each item scored such that socially desirable response increases the score by 1 point.

For BFI measures, the normality of residuals was violated as indicated by a significant Shapiro–Wilk test (W = 0.921–0.947, *p* < 0.001) for scales for Openness, Conscientiousness and Extraversion. We used Box-Cox transformation to find optimal lambda, then created squared scores in order to normalise them. While this intervention resolved the issue with Extraversion and Openness model, for Conscientiousness the residuals still deviated significantly from normality (W = 0.962, *p* < 0.05). As a result, for that model, robust regression using the *robustbase* package (Maechler et al., 2021) was implemented with original scores, which emerged as most parsimonious solution for addressing non-normality and improving the robustness of results (for more details on robustness checks, see Supplementary Table 2, p. 15).

**Figure 2:**
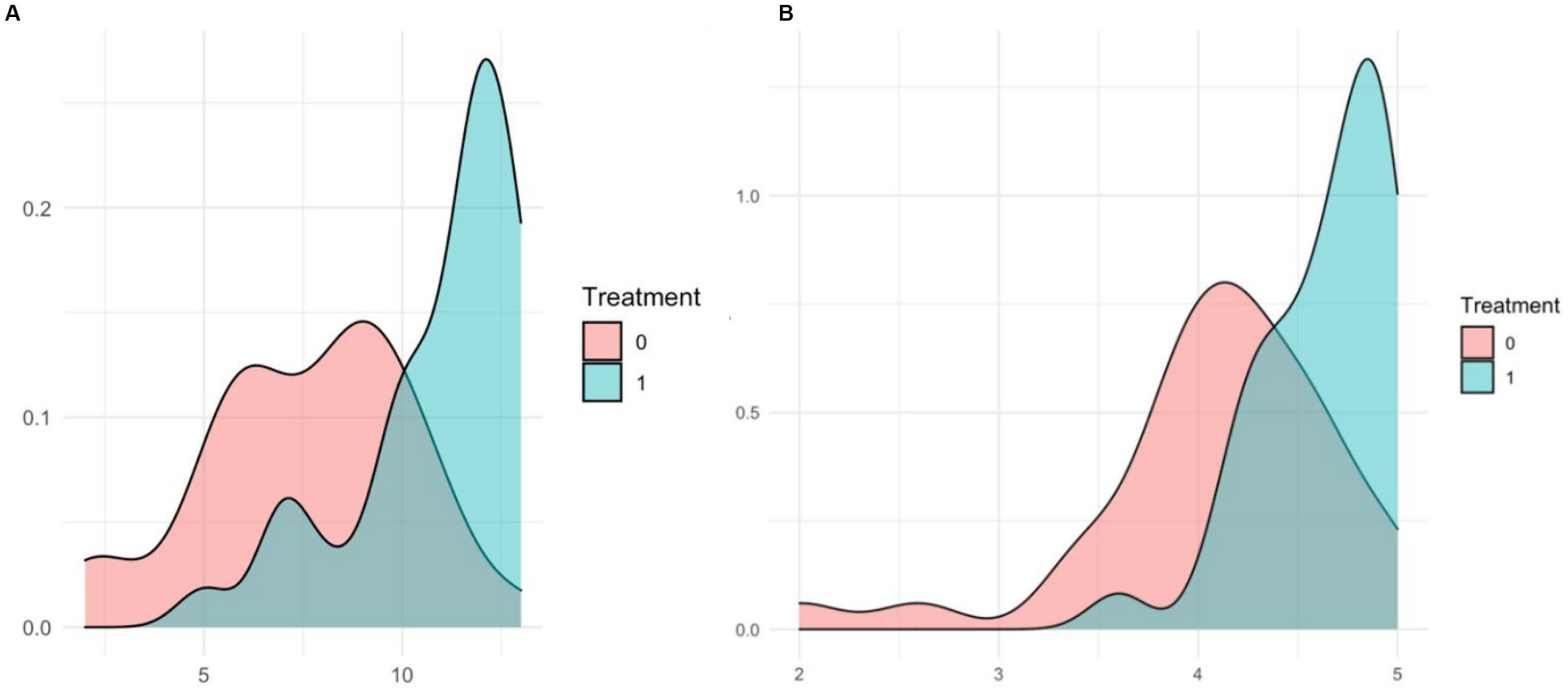
Density plots for scores on treatment group. **(A)** Distribution of MC scores. **(B)** Distribution of conscientiousness.

In the initial regression, professional selection significantly increases social desirability (MC) by 4.589 points (SE = 1.027, *p* < 0.001) and positively affects Openness by 0.448 points (SE = 0.597, *p* < 0.05), Conscientiousness by 0.460 points (SE = 0.214, *p* < 0.05), Extraversion by 0.605 points (SE = 0.212, *p* < 0.01), and Agreeableness by 0.650 points (SE = 0.274, *p* < 0.05). After applying HAC standard errors, the effect on MC remains significant at 4.553 points (SE = 1.203, *p* < 0.001) and on Extraversion at 0.640 points (SE = 0.173, *p* < 0.001). In the squared score models, we confirmed the robustness of this results as treatment remained significant for both Openness and Extraversion. Robust regression also confirmed the significant effect on Conscientiousness at 0.526 points (SE = 0.253, *p* < 0.05). Density plots of the distribution of MC (Figure 2A) and Conscientiousness (Figure 2B) scores by treatment group illustrate this significant difference and for details see Supplementary Table 7, p. 17.

#### 3.4.3 Regression analysis on chatbot inferred scores

To assess whether the AI-inferred traits are susceptible to social desirability bias, we replicated the analysis from segment 3.4.2., focusing solely on the AI-inferred traits. This analysis was conducted on a matched sample of 66 participants, selected based on propensity scores (see 3.4.1.). Assumptions checks, including tests for normality and homoscedasticity, revealed some deviations from across all five traits, as indicated by significant Shapiro–Wilk tests with *p*-values ranging from 0.0004 to 0.0233 (see Supplementary Table 2, p. 15).

Given these findings, we employed robust regression methods using the robustbase package (Maechler et al., 2021) to account for these violations. The analysis showed that the treatment variable, which indicates whether participants were in a professional selection context, did not have a significant effect on any of the five AI-inferred traits. Specifically, the p-values for the treatment variable across all models ranged from 0.304 to 0.997, consistently indicating non-significance (for details see Supplementary Table 8, p. 18). Results suggest that the AI-inferred traits are unlikely to be influenced by social desirability bias in professional selection scenarios. This finding supports Hypothesis 2, which posits that the chatbot, unlike traditional tests, is not susceptible to such bias.

## 4 Discussion

This study investigated how personality traits assessed via an AI-powered chatbot compared to traditional psychometric testing in a real-world hiring scenario. We implemented a novel *one-question-per-facet* approach and conducted the study in two non-WEIRD countries—Serbia and Montenegro—addressing a significant gap in the emerging field of AI-based personality inference, which has largely focused on English speaking population.

The results offered partial support for Hypothesis 1, indicating that AI-inferred personality traits showed acceptable structural and substantive validity, particularly for traits like Extraversion and Neuroticism. However, in line with prior research (Fan et al., 2023) issues emerged in areas such as discriminant validity, with Neuroticism and Agreeableness exhibiting significant overlap. Furthermore, the AI-inferred traits did not demonstrate strong criterion-related or predictive validity in real-world career outcomes, with traditional psychometric tests consistently outperforming AI in predicting education level and job position.

For Hypothesis 2, the results provided robust evidence that AI-inferred personality assessments are less susceptible to social desirability bias relative to traditional self-report psychometric tests, thus confirming the hypothesis. This finding highlights a significant advantage of chatbot-based assessments in minimising response distortions in selection contexts.

### 4.1 Theoretical and practical contributions

This study aimed to begin unpacking the “black box” of AI (Mills, 2021) in behavioural science and bridge the practitioner-academia gap in the field of AI Psychometrics (Will et al., 2022) by applying rigorous methodologies in a real-world professional selection scenario. It introduced three significant contributions to the existing literature: (1) using a practical, real-world hiring setting, (2) implementing a question-per-facet approach, and (3) exploring AI-driven psychometrics in an underrepresented language.

The findings confirm the replicability of the Five-Factor structure through AI-inferred personality scores, consistent with the results of Fan et al. (2023), Dai et al. (2022), and Grunenberg et al. (2024). These prior studies highlighted AI’s potential to infer personality traits with moderate alignment to traditional psychometric tests, particularly for Extraversion and Neuroticism. The adoption of a one-question-per-facet technique represents an advancement on Fan et al. (2023) framework, offering more granular insights into the psychometric properties of chatbot-inferred scores and addressing substantive validity challenges noted by Hickman et al. (2022).

However, this study diverges from prior findings in external validity. While Fan et al. (2023) and Grunenberg et al. (2024) reported incremental and criterion-related validity for AI-inferred scores in predicting academic performance and vocational interests, this research found no significant relationships between AI-inferred scores and career outcomes, such as education level or job role. This discrepancy likely reflects limitations in the sample and contextual factors, as Fan et al. (2023) utilised larger datasets in controlled environments, whereas this study examined real-world hiring scenarios in Serbia and Montenegro—a non-WEIRD context previously underexplored.

The findings on social desirability bias offer a significant contribution. Consistent with Fan et al. (2023), this research demonstrated that AI chatbots are resistant to social desirability effects, unlike traditional psychometric tests, which are prone to response distortion in high-stakes settings (Mikulić and Sekulić, 2014; Ziegler and Kemper, 2013). By validating AI methods in actual hiring scenarios, this study extends the ecological validity of prior findings, reinforcing the practical applicability of chatbots in reducing bias. This aligns with Dai et al.’s (2022) recommendation to validate AI methodologies in diverse and real-world contexts.

This study’s methodological innovation—introducing the one-question-per-facet approach—demonstrates how AI can address longstanding psychometric issues, such as improving substantive and structural validity, advancing methodological rigour, and offering a pathway for continuous improvement. By combining this methodological refinement with a high-stakes professional context, it bridges the practitioner-academia gap identified by Will et al. (2022). The real-world setting provided an opportunity to evaluate AI tools under actual hiring conditions, enhancing ecological validity and offering insights directly relevant to practical applications. Furthermore, conducting this research in an underrepresented linguistic and cultural context expands the applicability of AI-driven assessments, addressing an area that remains relatively unexplored.

From a practical perspective, the findings of this study hold significant implications for organisations adopting AI in recruitment. The demonstrated resistance of AI chatbots to social desirability bias highlights their potential in high stakes hiring scenarios where response distortion can compromise the accuracy of traditional assessments. Additionally, the ability to apply AI-driven psychometrics in diverse cultural and linguistic contexts expands their utility for global organisations seeking scalable, unbiased hiring solutions. By refining methodologies like the question-per-facet approach, this research provides a framework for organisations to enhance the reliability and validity of AI assessments, ensuring they meet both practical hiring needs and ethical standards.

While the findings partially replicate prior research, they uniquely emphasise the need for tailored validation in real-world applications. This highlights AI’s potential to enhance authenticity and reduce bias in personality assessments, contributing to the broader goal of refining AI-driven tools for robust and equitable hiring practises.

### 4.2 Limitations and future research recommendation

This study faced limitations, including the absence of an initial training phase (Fan et al., 2023) and a smaller sample size limited to the banking industry in candidate group. While these factors may limit the generalizability of results to broader populations, it is important to note that our sample size met the criteria recommended by power analyses and aligns with the recommendations provided by existing literature. Additionally, despite the absence of a training phase, the chatbot demonstrated good psychometric properties in many instances (as shown in Table 3) and was resistant to social desirability bias. This suggests that the fine-tuning approach using existing data and building on the extensive training of OpenAI’s ChatGPT 4.0 API was effective in achieving robust results.

Conducting a training study could have further improved the quality of the chatbot inference, especially in the context of under-represented language in which this method is conducted for the first time. However, the promising findings of this study show the feasibility and reliability of our approach even without an initial language-specific training phase.

To build on the present findings, future studies should include a larger and more diverse sample, which could strengthen the robustness and applicability of the results. Diversifying the candidate pool beyond a single industry would allow for broader generalisation and potentially increase the likelihood of detecting relationships with other criteria. Further development of the one-facet-per-question strategy could enhance chatbot validity. To deepen insights into content validity, future research should also examine test–retest reliability and work on further improving and evaluating facet questions by advancing this methodology. Additionally, this study showed that AI-derived scores are not susceptible to social desirability bias as self-report measures are. An intriguing direction for future research would be to explore whether AI can infer social desirability bias directly from text-based responses. Furthermore, it would be valuable to investigate whether human interviewers, following a similar judgement-based methodology as the chatbot, could infer personality traits from open-ended responses, potentially providing deeper insights despite being more labour-intensive.

## 5 Conclusion

In conclusion, this study highlights the potential of AI-inferred personality traits, particularly their resistance to response manipulation, while underscoring the need for further refinement and validation to match the rigour of psychometric measures. By advancing AI-driven psychometric methods and emphasising methodological rigour in real-world applications, this work contributes to the growing body of research at the intersection of AI and psychometrics, paving the way for more practical and scientifically robust personality assessments.

## Data Availability

The raw data supporting the conclusions of this article will be made available by the authors, without undue reservation.

## Footnotes

^1^https://sapia.ai/.

^2^ https://fabrile.app/

^3^ https://ingram.tech/

^4^ https://recrewty.com/
