# Supplementary material for: Comparing chatbots to psychometric tests in hiring: reduced social desirability bias, but lower predictive validity
Source: Front Psychol. 2025 Apr 25;16:1564979. doi: 10.3389/fpsyg.2025.1564979 (PMC12061966; doi:10.3389/fpsyg.2025.1564979)
Supplement: Supplementary file 1 [file Supplementary_file_1.docx]

**Supplementary materials**

1. **Materials and instruments**

*Note: All items were recoded, so they indicate a positive relationship with their corresponding trait.

***Note: In the Serbian short version of Big Five, Agreeableness scale is reversed as “Agressivity-Agreeableness”. To be consistency with prior research from literature in this area, this scale was recoded.

1. *Big Five plus Two: A short version (Colovic et al., 2014):*

Instructions: THIS QUESTIONNAIRE CONTAINS STATEMENTS THAT RELATE TO FEELINGS, OPINIONS, AND BEHAVIORS COMMON TO ALL PEOPLE. PLEASE CIRCLE THE NUMBER THAT BEST CORRESPONDS TO YOUR LEVEL OF AGREEMENT WITH THE GIVEN STATEMENT.

THE NUMBERS HAVE THE FOLLOWING MEANING:

1 - STRONGLY DISAGREE
2 - MOSTLY DISAGREE
3 - NOT SURE
4 - MOSTLY AGREE
5 - STRONGLY AGREE

| Original | Translated |
| --- | --- |
| Veoma sam uporan. | I am very persistent. |
| Pratim nova zbivanja u umetnosti (muzika, film, književnost…). | I follow new developments in art (music, film, literature...). |
| Družim se sa velikim brojem ljudi. | I socialize with a large number of people. |
| Lako se obeshrabrim. | I easily get discouraged. |
| Za mene važi: ono što možeš da uradiš danas, ne ostavljaj za sutra. | For me, the saying "what you can do today, don't leave for tomorrow" applies. |
| Imam različita interesovanja. | I have diverse interests. |
| Može se reći da sam prgava osoba. | It can be said that I am a quarrelsome person. |
| Volim ljude. | I love people. |
| Ja baš nemam sreće. | I am really unlucky. |
| Ja sam prijatna osoba. | I am a pleasant person. |
| Bavim se mnogim zanimljivim stvarima u slobodno vreme. | I engage in many interesting activities in my free time. |
| Veoma sam marljiv i vredan. | I am very diligent and hardworking. |
| Uvek ispunjavam sve svoje obaveze. | I always fulfill all my obligations. |
| Ja sam "teška" osoba. | I am a "difficult" person. |
| Često se osećam ogorčeno. | I often feel bitter. |
| Sve što počnem, to i završim. | I finish everything I start. |
| Volim da naređujem. | I like to give orders. |
| Osećam da je život nepravedan prema meni. | I feel that life is unfair to me. |
| Imam blagu narav. | I have a mild temper. |
| Ja sam nesrećna osoba. | I am an unhappy person. |
| Veoma sam temeljan u onome što radim. | I am very thorough in what I do. |
| Često me more tužne misli. | I am often plagued by sad thoughts. |
| Često protivrečim drugim ljudima. | I often contradict other people. |
| Sklon sam da odlažem obaveze. | I am prone to procrastination. |
| Pričljiv sam. | I am talkative. |
| Nemaran sam kada su obaveze u pitanju. | I am careless when it comes to obligations. |
| Često pobesnim. | I often get furious. |
| Lako se iznerviram. | I get irritated easily. |
| Uglavnom sam dobro raspoložen. | I am generally in a good mood. |
| Voleo bih da isprobam što više stvari u životu. | I would like to try as many things as possible in life. |
| Često sam zabrinut. | I am often worried. |
| Često se suprotstavljam mišljenju drugih. | I often oppose the opinions of others. |
| Osećam da mi nova saznanja obogaćuju život. | I feel that new knowledge enriches my life. |
| Lako planem. | I easily lose my temper. |
| Veoma sam srdačan. | I am very warm-hearted. |
| Neka umetnička dela mogu u meni da pobude snažna osećanja. | Some works of art can evoke strong emotions in me. |
| Stalno se usavršavam i napredujem. | I am constantly improving and progressing. |
| Ja sam vedra osoba. | I am a cheerful person. |
| Često me muči osećanje krivice. | I am often troubled by feelings of guilt. |
| Veoma sam društven. | I am very sociable. |
| Često tragam za informacijama o stvarima koje me zanimaju. | I often search for information about things that interest me. |
| Ja sam lenja osoba. | I am a lazy person. |
| Žudim za uzbuđenjima i novinama. | I crave excitement and new experiences. |
| Često mislim da život nema smisla. | I often think that life has no meaning. |
| Ja sam kreativna osoba. | I am a creative person. |
| Lako se zbližavam s ljudima. | I easily bond with people. |
| Često smandrljam neki posao. | I often rush through a job. |
| Često se posvađam sa drugima. | I often get into arguments with others. |
| Često osećam teskobu. | I often feel anxiety. |
| Pun sam energije. | I am full of energy. |

*Note: All items were recoded, so they indicate a positive relationship with their corresponding trait.

***Note: In the Serbian short version of Big Five, Agreeableness scale is reversed as “Agressivity-Agreeableness”. To be consistency with prior research from literature in this area, this scale was recoded.

1. *Marlowe-Crowne Social Desirability Scale (M-C SDS; Crowne & Marlowe, 1960; Reynolds, 1982)*

Instructions: Listed below are a number of statements concerning personal attitudes and traits. Read each item and decide whether the statement is true or false as it pertains to you.

| Original | Translated |
| --- | --- |
| Prije glasanja temeljno istražujem kvalifikacije svih kandidata. | Before voting I thoroughly investigate the qualifications of all the candidates. |
| Nikada ne oklijevam da pomognem nekome ko je u nevolji. | I never hesitate to go out of my way to help someone in trouble. |
| Ponekad mi je teško da nastavim sa radom ako nisam ohrabren. | It is sometimes hard for me to go on with my work if I am not encouraged. |
| Nikada nisam intenzivno mrzio nekoga. | I have never intensely disliked anyone. |
| Povremeno sumnjam u svoju sposobnost da uspijem u životu. | On occasion I have doubts about my ability to succeed in life. |
| Ponekad se osjećam ogorčeno kada ne dobijem ono što želim. | I sometimes feel resentful when I don't get my own way. |
| Uvijek pazim na svoj način oblačenja. | I am always careful about my manner of dress. |
| Moji maniri za stolom kod kuće su jednako dobri kao i kada jedem u restoranu. | My table manners at home are as good as when I eat out in a restaurant. |
| Ako bih mogao/la ući u bioskop bez plaćanja i bio siguran da me niko ne vidi, vjerovatno bih to uradio. | If I could get into a movie without paying and be sure I was not seen, I would probably do it. |
| Nekoliko puta sam odustao od nečega jer sam mislio da nisam dovoljno sposoban. | On a few occasions, I have given up doing something because I thought too little of my ability. |
| Ponekad volim da ogovaram. | I like to gossip at times. |
| Bilo je trenutaka kada sam osjećao potrebu da se pobunim protiv autoriteta iako sam znao da su u pravu. | There have been times when I felt like rebelling against people in authority even though I knew they were right. |
| Bez obzira s kim razgovaram, uvijek sam pažljivo slušam. | No matter who I’m talking to, I’m always a good listen |

*Note: All items were recoded, so they indicate a positive relationship with their corresponding trait.

1. *Chatbot questions (translated)*

Chatbot had data about traits and facets distribution based on Smederevac et al. (2014).

**Extraversion (Mean = 64.5; SD = 16.5)**

1. **Sociability (Mean = 62.25; SD = 22)**
   - "Describe a recent social event you attended. How did you feel while interacting with others, and how active were you in engaging with different people?" (This measures Sociability within Extraversion)
   - Follow-up questions:
     1. "Recall a time when you had to introduce yourself to strangers. How did you approach the situation, and what was your comfort level?"
     2. "Think about your friendships and social circles. How do you usually communicate with your friends, and what role do you play at social gatherings?"
     3. "Tell me about a situation when you decided whether or not to participate in a social activity. What influenced your decision, and how did you feel about it?"
2. **Assertiveness (Mean = 63; SD = 18.75)**
   - "Describe a situation where you had to express your opinion contrary to the majority. How did you handle that situation, and what was the outcome?" (This measures Assertiveness within Extraversion)
   - Follow-up questions:
     1. "Tell me about a time when you took on a leadership role in a group or project. How did you feel in that role, and how did others respond to your leadership?"
     2. "Can you share an experience when you decided not to let others make decisions for you? What motivated you, and how did you feel about it?"
     3. "Recall a situation where you had to influence others' decisions. How did you approach that situation, and how successful were you?"
3. **Energy Level (Mean = 68; SD = 20)**
   - "Can you describe a situation when you felt extremely excited and enthusiastic? What triggered those feelings, and how did you react?" (This measures Energy Level within Extraversion)
   - Follow-up questions:
     1. "Tell me about a day when you were particularly active and full of energy. What did you do that day, and how did you feel during all the activities?"
     2. "Share an experience when you displayed a lot of enthusiasm. How did others react to your enthusiasm, and how did you feel about it?"
     3. "Recall a time when you noticed that you were more active than the people around you. How did you feel in that situation, and what do you think contributed to your higher level of activity?"

**Agreeableness (Mean = 71.25; SD = 13.5)**

1. **Compassion (Mean = 74.75; SD = 17)**
   - "Can you describe a situation where you helped someone in distress? What motivated you to help, and how did you feel about it?" (This measures Compassion within Agreeableness)
   - Follow-up questions:
     1. "Tell me about a time when you felt deeply compassionate towards someone. What triggered those feelings, and how did you react?"
     2. "Share an experience when you offered support or comfort to someone. How did that person react, and how did you feel about your role?"
     3. "Recall a situation where you noticed someone was suffering or facing problems. How did you react, and what did you do to help them?"
2. **Respect (Mean = 77.5; SD = 15.75)**
   - "Can you describe a situation where you made an effort to show respect to someone, even when you disagreed with their opinion? How did you approach the situation, and how did the person react?" (This measures Respect within Agreeableness)
   - Follow-up questions:
     1. "Tell me about a time when you had the opportunity to be rude to someone but chose to be kind instead. What motivated you to act that way, and how did others react?"
     2. "Share an experience when you avoided an argument with someone, choosing instead to show understanding and respect. How did you feel about it, and how did it affect your relationship with that person?"
     3. "Recall a situation where you noticed that someone was not kind to you. How did you react, and how did you strive to remain polite?"
3. **Trust (Mean = 61.25; SD = 18.25)**
   - "Share an experience where you assumed someone was good, even though you didn't know them well. How did that person behave, and was your trust justified?" (This measures Trust within Agreeableness)
   - Follow-up questions:
     1. "Tell me about a time when you noticed that you were less suspicious of others' intentions than other people were. How did you feel in that situation, and what influenced your trust?"
     2. "Recall a situation where you decided not to look for faults in others but instead gave them a chance. How did you feel about it, and how did those people respond?"
     3. "Describe a time when you decided to trust someone, and how that trust affected your relationship with that person. What motivated you to trust them?"

**Conscientiousness (Mean = 68.25; SD = 16.5)**

1. **Organization (Mean = 72.75; SD = 21)**
   - "Can you describe how you usually organize your space at home or work? How important is it for you that things are in their place, and how do you feel when your space is tidy?" (This measures Organization within Conscientiousness)
   - Follow-up questions:
     1. "Tell me about a situation where you had to quickly clean up a mess. How did you handle it, and what did you do to make everything neat?"
     2. "Share an experience where you faced a disorganized environment. How did you react, and what did you do to bring order?"
     3. "Recall a time when you organized an event or project. How did you approach the organization, and how important was it for you that everything was systematic and orderly?"
2. **Productivity (Mean = 64; SD = 21)**
   - "Can you describe a situation where you managed to complete a large project or task on time? What motivated you, and how did you organize your work to be efficient?" (This measures Productivity within Conscientiousness)
   - Follow-up questions:
     1. "Tell me about a time when you faced a difficult task. How did you start working on it, and what motivated you to finish?"
     2. "Share an experience when you had to be very persistent to complete a job. How did you stay motivated, and what helped you not to give up?"
     3. "Describe a time when you had difficulty starting a task. How did you overcome those difficulties, and what eventually motivated you to complete the job?"
3. **Responsibility (Mean = 68; SD = 17.25)**
   - "Can you describe a situation where others relied on you to complete an important task? How did you approach the task, and how did others react to your work?" (This measures Responsibility within Conscientiousness)
   - Follow-up questions:
     1. "Tell me about a time when you had to take responsibility for a mistake you made. How did you handle the situation, and what did you do to correct it?"
     2. "Share an experience where you were responsible for an important project or task. How did you ensure that everything was done on time, and how did you strive to remain reliable?"
     3. "Describe a time when you had to demonstrate your reliability in a team or group. What did you do to ensure that everyone could count on you, and how did your contribution impact the team?"

**Neuroticism (Mean = 54.25; SD = 21)**

1. **Anxiety (Mean = 64.75; SD = 22.25)**
   - "Can you describe a situation when you felt very anxious or worried? What caused those feelings, and how did you cope with them?" (This measures Anxiety within Neuroticism)
   - Follow-up questions:
     1. "Tell me about a time when you were under a lot of stress. How did you react to the stress, and what did you do to reduce it?"
     2. "Share an experience where you felt tense or nervous. How did you deal with those feelings, and what helped you calm down?"
     3. "Recall a situation where you worried a lot about something. What caused your worry, and how did you manage those concerns?"
2. **Depressiveness (Mean = 45; SD = 25.5)**
   - "Can you describe a situation where you felt particularly sad or depressed? What caused those feelings, and how did you cope with them?" (This measures Depressiveness within Neuroticism)
   - Follow-up questions:
     1. "Tell me about a time when you faced failure. How did you react, and what was your level of optimism or pessimism afterward?"
     2. "Share an experience where you felt insecure about yourself. How did it affect your behavior, and what did you do to overcome that insecurity?"
     3. "Recall a period when you were depressed or very down. What caused those feelings, and how did you cope with them?"
3. **Emotional Stability (Mean = 53; SD = 24.25)**
   - "Can you describe a situation where your mood changed quickly? What caused that change, and how did you feel before and after?" (This measures Emotional Stability within Neuroticism)
   - Follow-up questions:
     1. "Tell me about a time when you felt very upset. What caused your distress, and how did you deal with those feelings?"
     2. "Share an experience where you had difficulty controlling your emotions. How did you handle the situation, and what did you do to calm down?"
     3. "Recall a situation where you felt very emotional or easily upset. How did you deal with those emotions, and how did others react to you?"

**Openness (Mean = 69; SD = 16.75)**

1. **Creative Imagination (Mean = 62.5; SD = 25.5)**
   - "Can you describe a situation where you came up with a creative solution to a problem? How did you come up with the idea, and how did you put it into action?" (This measures Creative Imagination within Openness)
   - Follow-up questions:
     1. "Tell me about a time when you had an original idea for a project or task. What inspired you, and how did you bring that idea to life?"
     2. "Share an experience where you were able to imagine something that others couldn't. How did you use your imagination, and what did you achieve with it?"
     3. "Recall a situation where you were full of new ideas. How did you develop those ideas, and how successful were you in implementing them?"
2. **Intellectual Curiosity (Mean = 60; SD = 26.75)**
   - "Tell me about a time when you engaged in an intellectual or philosophical discussion. What prompted you to get involved, and how did you feel during the discussion?" (This measures Intellectual Curiosity within Openness)
   - Follow-up questions:
     1. "Share an experience where you thought about a deep or abstract idea. What drew you to that idea, and how did you develop it further in your mind?"
     2. "Describe a time when you explored an abstract idea or concept. How did you approach that exploration, and what did you learn from the process?"
     3. "Can you describe a situation where you were very curious about a topic? How did you explore that topic, and what did you learn from it?"
3. **Aesthetic Sensitivity (Mean = 68; SD = 24)**
   - "Can you describe how art influences your life? How do you feel when you view paintings, listen to music, or read books?" (This measures Aesthetic Sensitivity within Openness)
   - Follow-up questions:
     1. "Tell me about a time when you were particularly impressed by a work of art (a painting, song, book, performance). What impressed you, and how did you react?"
     2. "Share an experience where you visited an art gallery, concert, or theater performance. What did you experience, and how did that experience affect you?"
     3. "Recall a situation where you discussed art with someone. What prompted the discussion, and what were your thoughts and feelings during the conversation?"

*Chatbot Log Sample (translated)*

**User:** Z2A3B4

**Chatbot:** Thank you! We will start with the questions.

Can you describe a recent social event you attended? How did you feel while interacting with others, and how active were you in engaging with different people?

**User:** I was at a conference, talked to many people. I felt very good and excited because everyone was in a good mood, my colleagues as well as the people I met.

**Chatbot:** Thank you for your response. Now I would like to ask you the following:

Describe a situation where you had to express your opinion contrary to the majority. How did you handle that situation, and what was the outcome?

**User:** We were working on a project in a consulting workshop where my colleagues wanted to address a problem with a PR campaign as a solution. From my experience, such interventions for that problem are not effective enough, so I proposed a solution based on economic incentives. Without any conflict, understanding that I had more experience than them in this area, I argued my position, and we agreed that my proposal would be the main intervention, and theirs would be supplementary.

**Chatbot:** Thank you for your detailed response. Now I would like to ask you the following:

Can you describe a situation where you felt extremely excited and enthusiastic? What triggered those feelings, and how did you react?

**User:** I was really excited recently before barbecuing with my best friend. We barbecued 8 kilograms of meat, socialized, joked around, the barbecue turned out phenomenal and met all our expectations.

**Chatbot:** Thank you for your response. Now I would like to ask you the following:

Can you describe a situation where you helped someone in need? What motivated you to help, and how did you feel about it?

**User:** I helped a friend when he lost his job by searching for jobs together and advising him even though I had a very busy schedule. I was also motivated by the fact that he is my friend, and I want the best for him.

**Chatbot:** Thank you for your response. Now I would like to ask you the following:

Can you describe a situation where you made an effort to show respect to someone, even when you disagreed with their opinion? How did you approach that situation, and how did the person react?

**User:** I recorded a podcast with a priest. We talked about the existence of God and the origin of morality. I am an evolutionary psychologist and do not believe in theological explanations, but that priest is a smart and good guy, so I approached the discussion with respect. Even though we have opposing views, the discussion was excellent, and we became friends afterward.

**Chatbot:** Thank you for your response. Now I would like to ask you the following:

Share an experience where you assumed someone was good, even though you didn't know them well. How did that person behave, and was your trust justified?

**User:** I always assume that someone is good. In the startup world we work in, people constantly meet and help each other. I could tell you 10 different examples where assuming someone was good paid off and created a positive outcome for me.

**Chatbot:** Thank you for your response. Now I would like to ask you the following:

Can you describe how you usually organize your space at home or work? How important is it for you that things are in their place, and how do you feel when your space is tidy?

**User:** My space is organized but not overly tidy. I like things to be in the same place, but I'm not overly concerned with cleaning or arranging things.

**Chatbot:** Thank you for your response. Now I would like to ask you the following:

Can you describe a situation where you managed to complete a large project or task on time? What motivated you, and how did you organize your work to be efficient?

**User:** I just finished a big report on time. I was motivated by the desire for my business to grow and to maintain a good relationship with investors.

**Chatbot:** Thank you for your response. Now I would like to ask you the following:

Can you describe a situation where others relied on you to complete an important task? How did you approach the task, and how did others react to your work?

**User:** I am the CEO of my startup, and others always count on me to do my part of the job. I approach it seriously, and I help others as they help me.

**Chatbot:** Thank you for your response. Now I would like to ask you the following:

Can you describe a situation where you felt very anxious or worried? What caused those feelings, and how did you cope with them?

**User:** I rarely feel anxious. The last time was when my girlfriend had a cold; I got a little worried about her health but quickly rationalized everything.

**Chatbot:** Thank you for your response. Now I would like to ask you the following:

Can you describe a situation where you felt particularly sad or depressed? What caused those feelings, and how did you cope with them?

**User:** I'm almost never sad. The last time was when my grandfather passed away, but I quickly turned those feelings of sadness into pride and happiness for the time we spent together.

**Chatbot:** Thank you for your response. Now I would like to ask you the following:

Can you describe a situation where your mood changed quickly? What caused that change, and how did you feel before and after?

**User:** I don't change my mood quickly. Most often, I am tired and irritable when my mood changes, and that would be when I become happy because I finished work and now my girlfriend is coming over.

**Chatbot:** Thank you for your response. Now I would like to ask you the following:

Can you describe a situation where you came up with a creative solution to a problem? How did you come up with the idea, and how did you put it into action?

**User:** I come up with creative solutions for my work every day, such as how to run a sales campaign or solve hiring problems for companies. Recently, I creatively solved the problem of redirecting respondents from a survey to a chatbot. I generally approach things analytically.

**Chatbot:** Thank you for your response. Now I would like to ask you the following:

Tell me about a time when you engaged in an intellectual or philosophical discussion. What prompted you to get involved, and how did you feel during the discussion?

**User:** I love philosophical discussions, and it's my favorite activity. I generally enjoy them and love to express my opinions and hear others'. For example, I often talk about the origin of morality and culture in relation to the history of peoples, with my colleague.

**Chatbot:** Thank you for your response. Now I would like to ask you the following:

Can you describe how art influences your life? How do you feel when you view paintings, listen to music, or read books?

**User:** I love reading books; it's one of my favorite hobbies. I read a lot in my field, but besides that, I enjoy Russian classics, Dostoevsky, etc.

**Assistant:** Thank you for all your responses. I will now provide feedback on your personality profile based on your answers.

Feedback for the CEO Position:

Strengths:

Your high extraversion and assertiveness make you an excellent leader who can communicate and motivate the team effectively.

High conscientiousness ensures that you are reliable and efficiently complete tasks, which is crucial for the success of your startup.

Your openness to new ideas and creative solutions helps you innovate and adapt to market changes.

Areas for Improvement:

Although you are emotionally stable, it is important to be aware of the emotional needs of your team and provide them with support when necessary.

Continue to develop your organizational skills to manage your time and resources even more effectively.

1. **Tables**

*Table 1: Descriptive statistics*

| Variable | M | SD | Min | Max | Skew | Kurtosis |
| --- | --- | --- | --- | --- | --- | --- |
| MC score | 8.54 | 2.86 | 1 | 13 | -0.29 | -0.69 |
| O | 4.14 | 0.46 | 2.60 | 5.00 | -0.69 | 0.62 |
| C | 4.19 | 0.60 | 2.00 | 5.00 | -0.96 | 0.79 |
| E | 4.05 | 0.55 | 1.90 | 5.00 | -0.47 | 0.28 |
| A | 2.77 | 0.67 | 1.00 | 4.50 | 0.58 | 0.55 |
| N | 1.85 | 0.63 | 1.00 | 4.30 | 1.03 | 1.40 |
| O (AI) | 73.73 | 11.21 | 35.00 | 91.67 | -0.93 | 0.94 |
| C (AI) | 80.47 | 8.92 | 53.33 | 92.33 | -0.99 | 0.43 |
| E (AI) | 75.73 | 12.31 | 18.33 | 90.00 | -1.43 | 2.35 |
| A (AI) | 76.17 | 9.18 | 43.33 | 90.00 | -0.91 | 0.84 |
| N (AI) | 57.57 | 14.12 | 20.00 | 80.00 | -0.90 | 0.12 |

*Note: M = Mean ; SD = Standard Deviation

**Note: For MC score, sum score was calculated, while for other variables, average score was derived

from their respective item/facet scores.

*Table 2: Results of assumptions checks for Regressions*

| Model | Shapiro-Wilk test  (W) | VIF (Highest Value) | Durbin-Watson test  (DW) |
| --- | --- | --- | --- |
| MC | 0.940** | 3.072 | 0.798*** |
| O | 0.942** | 3.072 | 2.140 |
| C | 0.921*** | 3.072 | 1.686 |
| E | 0.947** | 3.072 | 1.772 |
| A | 0.987 | 3.072 | 1.513 |
| O (AI) | 0.942** | 3.072 | 2.140 |
| C (AI) | 0.950** | 3.072 | 2.438 |
| E (AI) | 0.936** | 3.072 | 2.186 |
| A (AI) | 0.957* | 3.072 | 2.077 |
| N (AI) | 2.038 | 3.072 | 2.038 |

*Note: Significance levels are indicated as follows: *p < 0.05, **p < 0.01, ***p < 0.001.*

***Note:* Model Abbreviations: MC = Model Conscientiousness, O = Openness, C = Conscientiousness, E = Extraversion, A = Agreeableness, N = Neuroticism, AI = Artificial Intelligence. AI stands for AI inferred scores.

***Note: Significant Shapiro-Wilk test indicates deviations from normal distribution; VIF (Variance Inflation Factor): Values above 10 suggest high multicollinearity; Durbin-Watson Test: Values around 2 indicate no autocorrelation, values significantly lower suggest positive autocorrelation.

*Table 3: Results from regression of AI-inferred traits on Education level*

| Trait | Education Level | Coef. | Std. Error |
| --- | --- | --- | --- |
| O (AI) | Secondary Education | 0.03145 | 0.02179 |
|  | Higher Education | 0.05138 | 0.02374 |
| C (AI) | Secondary Education | 0.00468 | 0.02986 |
|  | Higher Education | 0.03896 | 0.03174 |
| E (AI) | Secondary Education | 0.01162 | 0.02039 |
|  | Higher Education | 0.02875 | 0.02139 |
| A (AI) | Secondary Education | 0.03144 | 0.02768 |
|  | Higher Education | 0.05182 | 0.02935 |
| N (AI) | Secondary Education | 0.01579 | 0.01902 |
|  | Higher Education | 0.00366 | 0.01993 |

***Note:* Model Abbreviations: MC = Model Conscientiousness, O = Openness, C = Conscientiousness, E = Extraversion, A = Agreeableness, N = Neuroticism, AI = Artificial Intelligence. AI stands for AI inferred scores.

*Table 4: Results from regression of AI-inferred traits on Career Outcomes*

| Trait | Coefficient | Std. Error | P-value |
| --- | --- | --- | --- |
| O (AI) | 0.01897 | 0.02019 | 0.347 |
| C (AI) | 0.01892 | 0.02540 | 0.456 |
| E (AI) | 0.01118 | 0.01723 | 0.516 |
| A (AI) | 0.01726 | 0.02423 | 0.476 |
| N (AI) | -0.00245 | 0.01604 | 0.879 |

***Note:* Model Abbreviations: MC = Model Conscientiousness, O = Openness, C = Conscientiousness, E = Extraversion, A = Agreeableness, N = Neuroticism, AI = Artificial Intelligence. AI stands for AI inferred scores.

*Table 5: Results from regression of traditional BFI and AI-inferred scores on Education level*

| Predictor | Coef. | Std. Error | Z-value | P-value | Significant |
| --- | --- | --- | --- | --- | --- |
| Extraversion | -1.387 | 0.793 | -1.748 | 0.0805 | No |
| Agreeableness | 0.658 | 0.598 | 1.101 | 0.2709 | No |
| Conscientiousness | 1.632 | 0.647 | 2.522 | 0.0117 | Yes |
| Neuroticism | -0.818 | 0.570 | -1.435 | 0.1512 | No |
| Openness | 1.476 | 0.819 | 1.803 | 0.0713 | No |
| Extraversion (AI) | 0.010 | 0.034 | 0.301 | 0.7635 | No |
| Agreeableness (AI) | 0.025 | 0.041 | 0.602 | 0.5470 | No |
| Conscientiousness (AI) | -0.018 | 0.047 | -0.371 | 0.7105 | No |
| Neuroticism (AI) | -0.015 | 0.025 | -0.589 | 0.5560 | No |
| Openness (AI) | 0.037 | 0.034 | 1.101 | 0.2709 | No |
| Age | -0.011 | 0.034 | -0.328 | 0.7426 | No |
| Gender | -0.449 | 0.714 | -0.628 | 0.5300 | No |
| Treatment | -2.595 | 0.893 | -2.906 | 0.0037 | Yes |

*Table 6: Results from regression of traditional BFI and AI inferred scores on Career outcomes*

| Predictor | Coef. | Std. Error | P-value |
| --- | --- | --- | --- |
| (Intercept) | -7.726 | 3.989 | 0.053 |
| Extraversion | 0.146 | 0.485 | 0.763 |
| Agreeableness | -0.089 | 0.369 | 0.810 |
| Conscientiousness | 0.058 | 0.438 | 0.895 |
| Neuroticism | -0.046 | 0.451 | 0.918 |
| Openness | 1.130 | 0.655 | 0.084 |
| Extraversion (AI) | -0.007 | 0.025 | 0.768 |
| Agreeableness (AI) | -0.011 | 0.034 | 0.737 |
| Conscientiousness (AI) | 0.030 | 0.037 | 0.425 |
| Neuroticism (AI) | -0.012 | 0.018 | 0.496 |
| Openness (AI) | 0.009 | 0.028 | 0.741 |
| Age | 0.057 | 0.023 | 0.015 * |
| Gender | -0.749 | 0.522 | 0.151 |
| Treatment | -19.720 | 1511.000 | 0.990 |

*Table 7: Results from regression of treatment on Psychometric test scores*

|  | (1)  MC | (2)  O. | (3)  C. | (4)  E. | (5)  A. | (6)  N. |
| --- | --- | --- | --- | --- | --- | --- |
| Panel A: Regression | | | | | | |
| Professional  Selection | 4.589***  (1.027) | 0.448*  (0.597) | 0.460*  (0.214) | 0.605**  (0.212) | 0.650*  (0.274) | -0.218  (0.247) |
| constant | 3.183  (3.091) | 2.812***  (0.597) | 3.558***  (0.646) | 3.586***  (0.638) | 3.176***  (0.274) | 2.201**  (0.745) |
| Panel B: HAC Standard Errors | | | | | | |
| Professional  Selection | 4.553***  (1.203) |  |  |  | 0.640***  (0.173) |  |
| constant | 3.550*  (1.745) |  |  |  | 3.066***  (0.679) |  |
| Panel C: Regression with squared dependent variables | | | | | | |
| Professional  Selection |  | 3,158*  (1.552) |  | 4.774**  (1.630) |  |  |
| constant |  | 8.110  (3.158) |  | 13.131**  (4.906) |  |  |
| Panel D: Robust regression | | | | | | |
| Professional  Selection |  |  | 0.526*  (0.253) |  |  |  |
| constant |  |  | 3.984***  (0.502) |  |  |  |

*Note: Marlowe-Crowne social desirability bias (MC) and the Big Five Inventory (BFI) traits: openness (O), conscientiousness (C), extraversion (E), agreeableness (A), and neuroticism (N). Standard errors are reported in parentheses. Significance Levels: *p < .05; **p < .01; ***p < .001. All models include controls for gender, age, education, job position, and industry type. The sample size for all models is 66.

*Table 8: Results from robust regression of treatment on AI-inferred scores*

|  | (2)  O. | (3)  C. | (4)  E. | (5)  A. | (6)  N. |
| --- | --- | --- | --- | --- | --- |
| Professional Selection | -0.133  (4.643) | 0.007  (1.976) | -2.405  (2.702) | 1.817  (1.754) | 2.675  (10.236) |
| constant | 73.268***  (12.897) | 83.436***  (7.765) | 91.401***  (22.213) | 77.777***  (9.606) | 32.740  (24.985) |

*Note: Marlowe-Crowne social desirability bias (MC) and the Big Five Inventory (BFI) traits: openness (O), conscientiousness (C), extraversion (E), agreeableness (A), and neuroticism (N). Robust standard errors are reported in parentheses. Significance Levels: *p < .05; **p < .01; ***p < .001. All models include controls for gender, age, education, job position, and industry type. The sample size for all models is 66.

**3***.* **Figures**

*Figure 1: AI-inferred scores distribution – Openness*

**
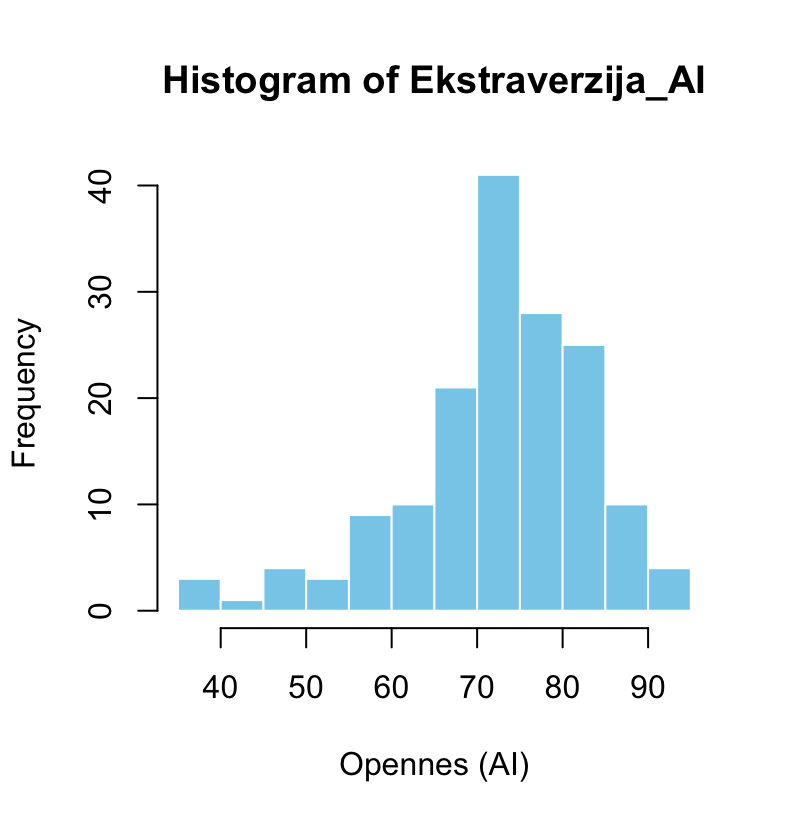
**

*Figure 2: AI-inferred scores distribution – Conscientiousness*

**
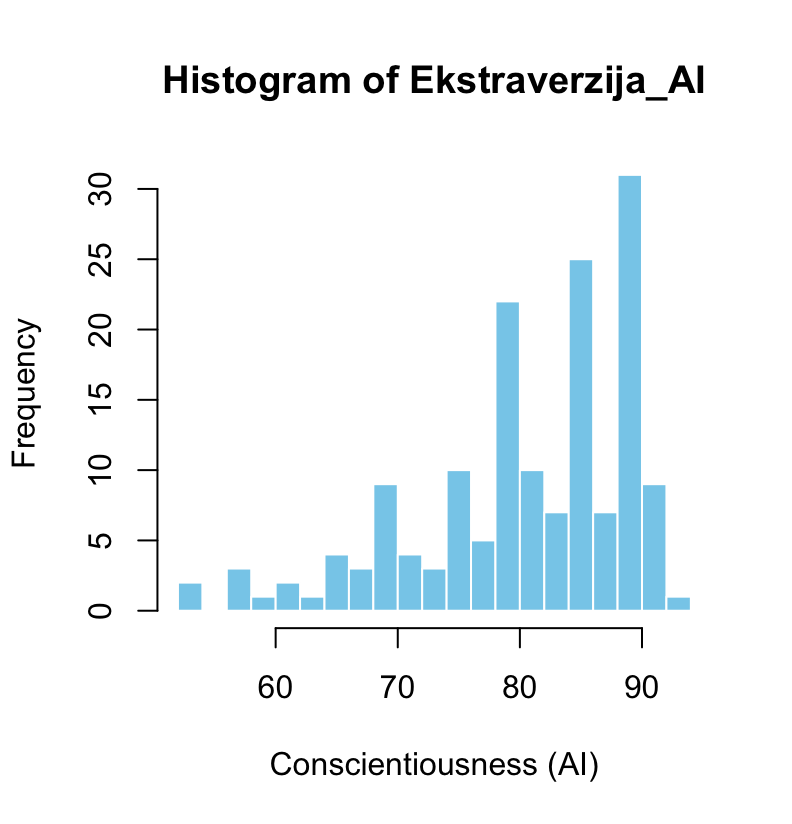
**

*Figure 3: AI-inferred scores distribution – Extraversion*

*
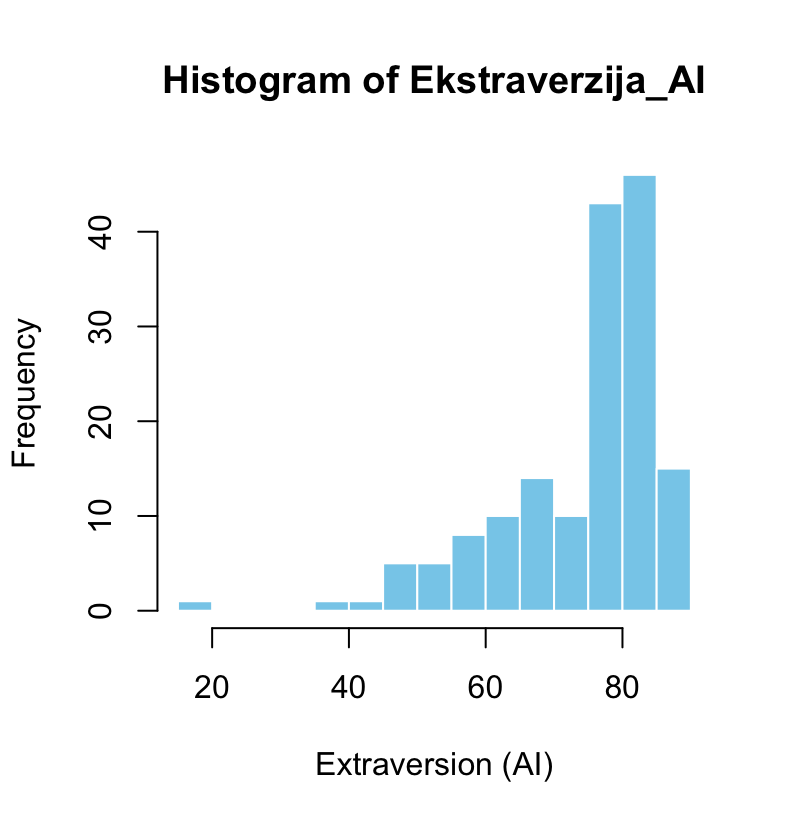
*

*Figure 4: AI-inferred scores distribution – Agreeableness*

*
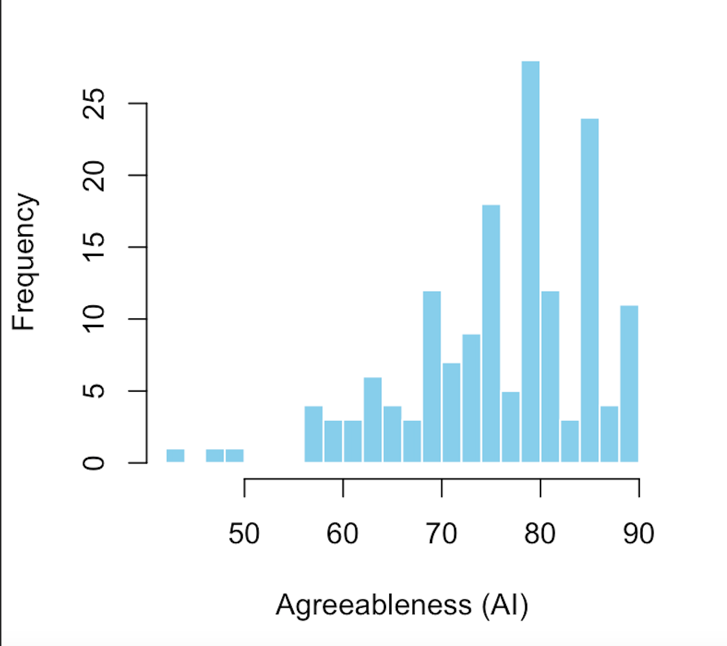
*

*Figure 5: AI-inferred scores distribution – Neurotiscism*

*
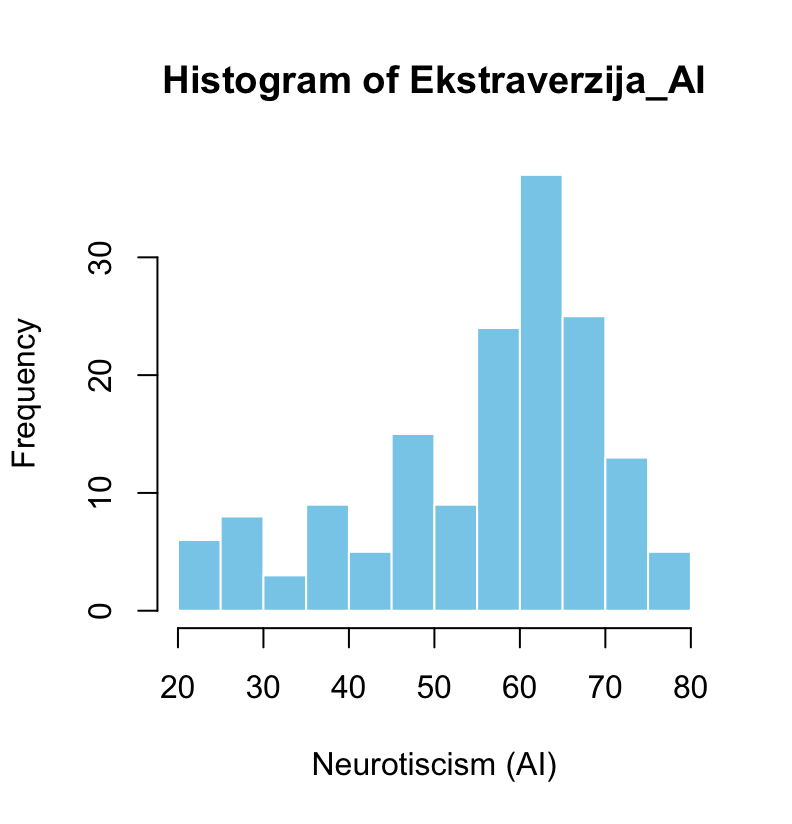
*
